# Nanopore Whole Transcriptome Analysis and Pathogen Surveillance by a Novel Solid‐Phase Catalysis Approach

**DOI:** 10.1002/advs.202103373

**Published:** 2021-11-27

**Authors:** Yi Fang, Amogh Changavi, Manyun Yang, Luo Sun, Aihua Zhang, Daniel Sun, Zhiyi Sun, Boce Zhang, Ming‐Qun Xu

**Affiliations:** ^1^ New England Biolabs, Inc. Ipswich MA 01938 USA; ^2^ Department of Microbiology and Immunology Cornell University Ithaca NY 14853 USA; ^3^ Department of Food Science and Human Nutrition University of Florida Gainesville FL 32603 USA

**Keywords:** direct RNA‐seq, foodborne pathogen, immobilized enzymes, next‐generation sequencing, Oxford Nanopore Technologies, transcriptome

## Abstract

The requirement of a large input amount (500 ng) for Nanopore direct RNA‐seq presents a major challenge for low input transcriptomic analysis and early pathogen surveillance. The high RNA input requirement is attributed to significant sample loss associated with library preparation using solid‐phase reversible immobilization (SPRI) beads. A novel solid‐phase catalysis strategy for RNA library preparation to circumvent the need for SPRI bead purification to remove enzymes is reported here. This new approach leverages concurrent processing of non‐polyadenylated transcripts with immobilized poly(A) polymerase and T4 DNA ligase, followed by directly loading the prepared library onto a flow cell. Whole transcriptome sequencing, using a human pathogen* Listeria monocytogenes* as a model, demonstrates this new method displays little sample loss, takes much less time, and generates higher sequencing throughput correlated with reduced nanopore fouling compared to the current library preparation for 500 ng input. Consequently, this approach enables Nanopore low‐input direct RNA‐seq, improving pathogen detection and transcript identification in a microbial community standard with spike‐in transcript controls. Besides, as evident in the bioinformatic analysis, the new method provides accurate RNA consensus with high fidelity and identifies higher numbers of expressed genes for both high and low input RNA amounts.

## Graphical abstract

High input requirement in Nanopore direct RNA‐seq is attributed to significant sample loss associated with library purification using solid‐phase reversible immobilization (SPRI) beads. This investigation employs immobilization of poly(A) polymerase and T4 DNA ligase to magnetic microbeads for RNA library preparation to circumvent SPRI bead purification. This approach enables low input transcriptomic analysis and improves sensitivity of pathogen detection.

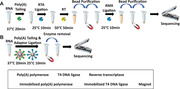

## 1 Introduction

High‐throughput RNA sequencing (RNA‐seq) techniques have transformed biological and biomedical research facilitating the understanding of transcriptional regulatory networks and identifying diagnostic and prognostic biomarkers for numerous diseases.^[^ ^1^ ^]^ The third‐generation sequencing technology offered by Oxford Nanopore Technologies (ONT) enables the sequencing of long reads, and most importantly, RNA molecules.^[^ ^2^ ^]^ Nanopore direct RNA‐seq avoids the artifacts and biases associated without amplification process.^[^ ^2^, ^3^ ^]^ Enabling the interrogation of native transcripts has inspired new ideas in RNA research, as evidenced by recent studies that elucidate transcriptome complexity from transcript isoforms and strand‐specific information that includes RNA base modifications.^[^ ^4^ ^]^ In addition, Nanopore sequencing using the portable MinION device allows for point of care, real‐time identification, and characterization of RNA viruses such as Ebola virus,^[^ ^5^ ^]^ Venezuelan equine encephalitis virus,^[^ ^5b^ ^]^ Zika virus,^[^ ^6^ ^]^ influenza A virus,^[^ ^7^ ^]^ and most recently, SARS‐CoV‐2.^[^ ^8^ ^]^ Furthermore, it allows for a single sequencing reaction to detect multiple, single‐stranded RNA viruses.^[^ ^9^ ^]^

Nanopore direct RNA‐seq will continue to grow in popularity with further improvement to sequencing chemistry, library construction methodology, and data processing pipelines. For instance, lowering the RNA input requirement can improve the robustness of transcript profiling and identification, provide increased sensitivity to identify single nucleotide polymorphisms , as well as facilitate early and sensitive detection of pathogenic bacteria and RNA viruses. In addition, quantification of gene expression and characterization of RNA biomarkers require unbiased coverage across the transcriptome. Hence, RNA‐seq based research relies on a high‐fidelity library preparation that can represent the entire transcriptome (the original RNA population) so that the RNA‐seq data can reflect genuine biological effects. It is well known that sequencing chemistry and bioinformatics tools can alter the final outcomes in next‐generation sequencing (NGS).^[^ ^10^ ^]^ NGS library construction, however, can cause specific biases.^[^ ^10^, ^11^ ^]^ It is of great interest to identify the technical and methodological artifacts that may introduce biases and affect enzymatic efficiency and offer viable solutions to improve the Nanopore RNA‐seq outcomes.

**Figure 1:**
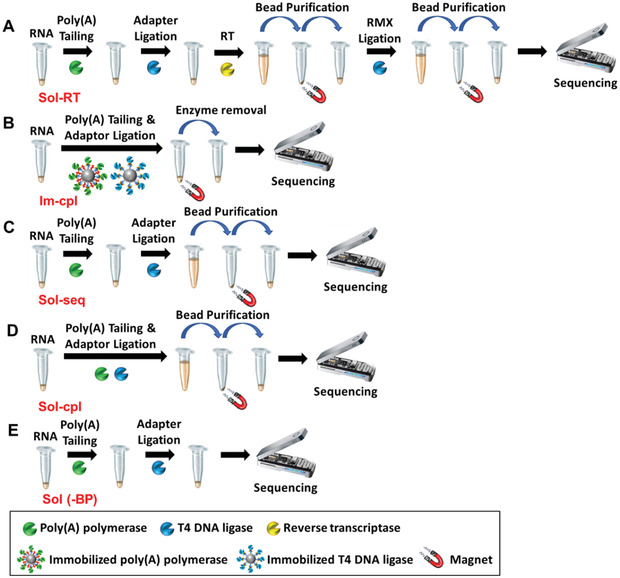
Schematic illustration of flowcharts for various library preparation protocols. A) Standard protocol using soluble enzymes with a reverse transcription step (Sol‐RT) and two bead‐based purification steps. B) Protocol using immobilized enzymes for coupled enzymatic reactions (Im‐cpl) without bead‐based purification. C) Protocol using soluble enzymes for sequential reactions (Sol‐seq) with single bead‐based purification. D) Protocol using soluble enzymes for coupled enzymatic reactions (Sol‐cpl) with single bead‐based purification. E) Protocol using soluble enzymes for sequential reactions without bead‐based purification [Sol (‐BP)].

We previously exploited Nanopore direct RNA‐seq for multiplex identification of viable pathogenic bacteria,^[^ ^12^ ^]^ including *Listeria monocytogenes*, the etiological agent of listeriosis, which has one of the highest case fatality rates among all foodborne illnesses.^[^ ^13^ ^]^ The current protocol demands high RNA input (500 ng), which hinders the implementation of this technique for biomedical research using low input amounts and sensitive pathogen surveillance as a point‐of‐care platform. The protocol for polyadenylated transcripts includes enzymatic addition of adaptor(s) and multiple purification steps with solid‐phase reversible immobilization (SPRI) beads (Figure S1, Supporting Information).^[^ ^12^ ^]^ Eukaryotic messenger RNA is mostly 3′ adenylated, which can be ligated directly to a reverse transcription adaptor (RTA) in the presence of T4 DNA ligase. The RNA–RTA hybrid products are then reverse transcribed in the presence of reverse transcriptase. The synthesis of a cDNA strand can improve throughput, presumably by destabilizing RNA secondary structures.^[^ ^2^ ^]^ Next, the reverse‐transcribed RNA–RTA complex is ligated to a second adaptor (RMX) preloaded with a motor protein required to guide the RNA strand through a nanopore channel in a flow cell. Additionally, 3′ polyadenylation is required prior to adaptor ligation (**Figure** **1**A), in cases where non‐polyadenylated RNA is to be sequenced, such as ribosomal RNAs (rRNAs) generated by RNA polymerase I and III, other small RNAs generated by RNA polymerase III, certain subsets of histone mRNAs, and long noncoding RNAs.^[^ ^14^ ^]^

Over the past decade, SPRI paramagnetic bead‐based chemistry has been widely used for nucleic acid handling procedures such as NGS library construction. While providing convenience in the removal of enzymes and other components, SPRI bead‐based purification can result in a fairly substantial sample loss, and thus researchers are required to begin their library preparation with large amounts of RNA input.^[^ ^2^, ^9^ ^]^ Besides, the sample loss can potentially introduce biases into the final prepared library. In addition, the extensive application of bead‐based purification steps also results in an extended library preparation time (about 3 to 4 h) before RNA‐seq. Furthermore, the current library preparation method comprises several distinct enzymatic reactions, resulting in a laborious and time‐consuming workflow. Therefore, it is highly desirable to improve the efficiency in library preparation and reduce sample loss inherent with SPRI bead‐based purification to enable Nanopore direct RNA‐seq viable for small input quantities or rare, difficult‐to‐obtain samples.

In this study, we intended to combine molecular enzymology and biochemical engineering approaches to develop a highly robust method to prepare a non‐poly(A) RNA library for Nanopore direct RNA‐seq using *L. monocytogenes* as a model. We exploited the immobilization of poly(A) polymerase (PAP) and T4 DNA ligase on magnetic microbeads as a means to achieve enzyme removal (Figure 1B), as opposed to SPRI‐based nucleic acid purification. We show that magnetic removal of the immobilized enzymes significantly reduced sample loss, thereby enabling Nanopore direct RNA‐seq analysis from significantly lower input amounts. Moreover, we validated a coupled reaction scheme to allow for a single‐tube RNA library preparation reducing the handling time from 3 to 4 h using the current protocol to slightly over 30 min. This solid‐phase catalysis strategy enabled highly efficient library preparations for a wide range of input RNA amounts, producing high throughput and transcript coverage, lower nanopore fouling rate, and a more complete transcriptome capture when compared to the current protocol using soluble enzymes. We achieved direct metatranscriptome sequencing using RNA amounts, more than an order of magnitude lower than the current input requirement.

## 2 Results

### 2.1 Design of Solid‐Phase Catalysis and Coupled Enzymatic Reaction Strategies

In this study, we investigated two new strategies for enzymatic processing of non‐polyadenylated *L. monocytogenes* RNA for Nanopore direct RNA‐seq (Figure 1). First, we evaluated a coupled reaction protocol (cpl) by the design of the concurrent presence of both poly(A) polymerase and T4 DNA ligase to allow coupling poly(A) tailing and adaptor ligation. We attempted to determine whether this coupled reaction strategy can improve the efficiency of library preparation. This new strategy was compared to the sequential reaction (seq) approach that resembles the current ONT library preparation workflow with 3′ poly(A) tailing and adaptor ligation reactions performed in two or more distinct steps. In addition, we thought to circumvent the significant sample loss due to bead‐based purification by using enzymes immobilized to magnetic microbeads to substitute their soluble form. We devised a coupled reaction protocol using immobilized enzymes, termed Im‐cpl, and compared it to the two soluble enzyme protocols. Purification using RNAClean XP beads was performed as the final clean‐up step for the sequential and coupled reaction protocols using soluble poly(A) polymerase and T4 DNA ligase, termed Sol‐seq and Sol‐cpl, respectively. A sequential reaction protocol using soluble enzymes without a clean‐up step (Sol (‐BP)) was also carried out. We previously showed that T4 DNA ligase covalently immobilized as SNAP‐tagged fusion protein onto O^6^‐benzylguanine (BG)‐coated magnetic microbeads (Im‐Ligase) can be utilized to catalyze a variety of ligation reactions.^[^ ^15^ ^]^ In this work, we demonstrate that this immobilized T4 DNA ligase catalyzes efficient ligation of two ONT adaptor‐derived substrates and can be easily removed from the reaction mixture (Figure S2, Supporting Information). We further achieved the generation of functional SNAP‐tagged poly(A) polymerase (Im‐PAP) by covalently conjugation onto magnetic microbeads modified with both BG ligand and polyethylene glycol polymer (Figure S3, Supporting Information). We have characterized 3′ poly(A) tailing activity and stability of Im‐PAP in comparison with its tagged and untagged soluble form (Figures S4 and S5, Supporting Information). Both soluble and immobilized PAP enzymes exhibit essentially the same processivity for 3′ poly(A) tailing (Figure S6, Supporting Information). The studies based on synthetic RNA oligomer indicate that it is feasible to use the immobilized PAP and T4 DNA ligase to process RNA library preparation for Nanopore direct RNA‐seq (Figure S7, Supporting Information). This strategy would omit the typical bead‐based clean‐up by pelleting the immobilized enzymes on a magnetic rack before transferring a prepared RNA library to a flow cell for a sequencing run. We conducted poly(A) tailing treatment of *L. monocytogenes* RNA with soluble PAP followed by RNAClean XP bead purification, or immobilized PAP followed by collecting the reaction medium after pelleting the enzyme. Indeed, we observed essentially no sample loss using the Im‐PAP compared to more than 50% sample loss using RNAClean XP bead purification (Figure S8, Supporting Information).

### 2.2 Comparison of Sequencing Yields of Different RNA Library Preparation Protocols

**Figure 2:**
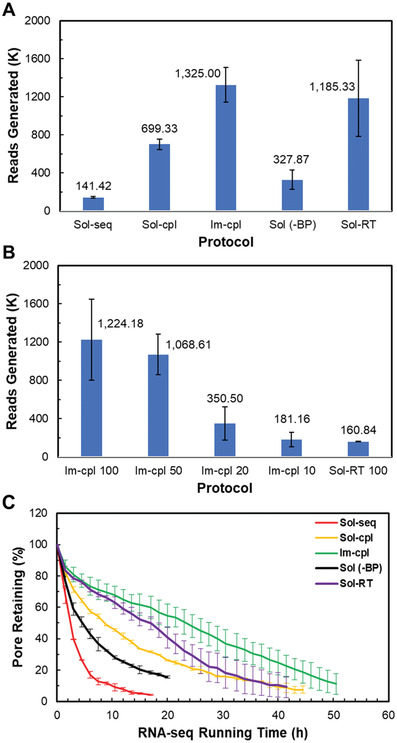
Direct RNA reads and nanopore fouling rate. A) Direct RNA reads generated from single flow cell run of each *L. monocytogenes* total RNA library using various protocols as depicted in Figure 1. B) Reads generated with low RNA input from 100, 50, 20, and 10 ng. 100 ng of RNA input was also used for soluble enzyme with a reverse transcription step (Sol‐RT 100). C) Nanopore fouling demonstrated by nanopore retaining percentage plotted against flow cell run time using various library preparation protocols.

We prepared the replicate RNA libraries using various protocols depicted in Figure 1, each with an input amount of 500 ng *L. monocytogenes* total RNA. The soluble enzyme protocols that include RNAClean XP purification as a clean‐up step (i.e., Sol‐seq and Sol‐cpl) showed a significant sample loss with an average recovery rate of 34% (**Table** **1**). Each Sol‐seq and Sol‐cpl library following a single bead‐based purification step was used for a sequencing run on a MinION R9.4 flow cell. Interestingly, the Sol‐cpl protocol produced more sequencing reads (an average of 699.3 K) than the Sol‐seq protocol (141.4 K reads) (**Figure** **2**), We further examined the recommended protocol, carried out using soluble PAP and T4 DNA ligase with a reverse transcription step using Superscript III. This protocol, Sol‐RT included two bead‐based clean‐up steps, and the library preparations suffered more sample loss compared to the Sol‐seq and Sol‐cpl protocols. However, sequencing the entire library loaded on a single flow cell yielded an average of 1.18 million (M) reads, significantly higher than the sequencing outputs from the two soluble enzyme protocols without reverse transcription described above. By contrast, the protocol that utilized solid‐phase enzymes (Im‐cpl) had essentially no sample loss, and the prepared Im‐cpl library, containing ≈500 ng RNA in a total volume of 40 µL, was available for sequencing on multiple flow cells (Table 1). When approximately one‐third of each prepared Im‐cpl library was loaded on a single flow cell, these sequencing runs generated an average of 1.32 M reads per flow cell, similar to the sequencing read output of the Sol‐RT but much higher than the Sol‐Seq and Sol‐cpl sequencing yields with similar read qualities (Figure 2 and Table 1). One of the Im‐cpl libraries was entirely loaded (40 µL) to three R9.4 flow cells, and the sequencing runs generated a total of 2.56 M reads, considerably higher than the read output acquired with a typical Sol‐RT library in this study (Table 1). Furthermore, in order to validate the necessity of enzyme removal before loading onto a flow cell, duplicate libraries using the sequential reaction protocol without bead purification [Sol (‐BP)] (Figure 1) were also performed. Because this protocol did not involve a bead‐based clean‐up step, no RNA sample loss was observed (Table 1); a single flow cell sequencing run, however, averaged 327.9 K reads, much lower than the Im‐cpl protocol (Figure 2), suggesting that retaining enzymes have a negative effect of on Nanopore sequencing performance.

**Table 1:** Comparison of library metrics of Nanopore direct RNA‐seq using soluble or immobilized enzymes by sequential or coupled enzymatic protocols using 500 ng of RNA input

	RNA input: 500 ng					
	Bead‐based purification			No bead‐based purficaiton		
	Sol‐seq	Sol‐cpl	Sol‐RT	Im‐cpl (1/3)	Im‐cpl	Sol (‐BP)
Recovery rate^a)^ [%]	34	34	24	106	131	100
Loading (ng)^b)^	186.4	208.8	112.0	190.3	623.2	164.4
Loading [µL]	20^c)^	20^c)^	20^c)^	13.4^d)^	38^d)^	10^d)^
Loading percentage [%]	100	100	100	34	100	25
Reads generated [K]/flow cell	141.4	699.3	1185.3	1325.0	–	327.9
Reads generated [K]/library^e)^	141.4	699.3	1185.3	–	2566.6	327.9
RNA‐seq time [h]^f)^	20.2	48.8	53.0	65.8	–	31.8
Mean read quality	10.2	10.1	10.3	10.2	–	10.3
Median read quality	10.4	10.2	10.4	10.2	–	10.5
Mean read quality (‐rRNA^g)^)	9.2	9.0	–	9.1	–	9.1
Median read quality (‐rRNA^g)^)	9.2	8.8	–	8.8	–	8.9

^a)^ Recovery rate measures the percentage of RNA before and after RNA library preparation (performed with a minimum of two replicates) by each method.

^b)^ The entire prepared libraries generated by the soluble enzyme protocols, i.e., Sol‐seq, Sol‐cpl, Sol‐RT were loaded on a single flow cell. One‐third of the sample was loaded on a single flow cell for Im‐cpl (1/3), and the entire library was split to three parts and loaded on three flow cells for Im‐cpl. Total of 20 µL collected after bead‐based purification was loaded on a MinION R9.4 flow cell.

^c)^ Total of 20 µL collected after bead‐based purification was loaded on a MinION R9.4 flow cell.

^d)^ Due to the volume capacity of the library loading to each flow cell, the maximum volume of loading was 15 µL as we tested. The amount of each Im‐cpl (1/3) and Sol (‐BP) library per sequencing run was comparable to that of Sol‐seq. The entire RNA library, except for 2 µL, was loaded for Im‐cpl.

^e)^ All protocols generated a mapped rate of ≈99% before the removal of rRNA sequences.

^f)^ RNA‐seq time refers to the time RNA‐seq continues until the flow cell retains less than 10 active pores.

^g)^ rRNA were removed in the data analysis.

### 2.3 Nanopore Fouling by Various Protocols

Interestingly, we noticed a correlation between the sequencing yield and flow cell run time from different protocols (Figure 2). ONT R9.4 flow cell contains up to 512 nanopore channels for sequencing DNA or RNA in real‐time. During each sequencing run, the pore complex tends to be deteriorated and become inactive. We found that different protocols can profoundly affect the sequencing time or the decay of the nanopore sensors. The low data output from the soluble enzyme protocol without bead purification (Sol (‐BP)) and the sequential reaction protocol using soluble enzymes (Sol‐seq) (shown in Figure 2A) appear to correlate with fast nanopore fouling rate, and the shorter sequencing runs less than 24 h (Figure 2C). On the other hand, both coupled reaction protocols (Sol‐cpl and Im‐cpl) displayed higher output and longer sequencing time than the sequential reaction protocols (Sol‐seq). The Sol‐RT protocol improved run time compared to the Sol‐cpl and Sol‐Seq protocols, albeit a slightly shorter run time than the Im‐cpl protocol. The Im‐cpl protocol exhibited the longest average run time of 65.8 hours, consistent with its high read output. Thus, more reads and bases generated from the sequencing runs using the immobilized enzymes can be attributed to the longer functioning life of the nanopores.

### 2.4 Solid‐Phase Catalysis Enables Low‐Input RNA Direct Sequencing

Given the high recovery rate and improved sequencing throughput of the coupled reaction protocol using the immobilized enzymes (Im‐cpl), we further conducted a series of library preparations from lower inputs of *L. monocytogenes* RNA for direct RNA sequencing. We set up the low input library preparation using the immobilized enzymes in 15 µL volume reactions so that the prepared library can be entirely applied to a single flow cell. We began with a comparative analysis of the libraries from 100 ng RNA inputs using the Im‐cpl and Sol‐RT protocols. We noticed essentially no sample loss using the Im‐cpl protocol to process 100 ng of *L. monocytogenes* RNA input (Im‐cpl 100) and a substantial loss (recovery rate of 37%) using the Sol‐RT 100 protocol with a bead purification step. The Im‐cpl protocol generated an average of 1.22 M reads from the replicates (Figure 2B). By contrast, the replicates of the 100 ng input libraries prepared by the Sol‐RT protocol yielded an average of 160.8 K reads, indicating the adverse effect of significant RNA sample loss during sample preparation. We further explored library preparations with lower RNA inputs (i.e., 50, 20, and 10 ng) using the same Im‐cpl protocol formulated for low input RNA. With 50 ng RNA input, we observed an average recovery of 92.1% with the use of immobilized enzymes. RNA recovery rate was not measured for RNA input of 20 ng and below because the RNA content was below the Qubit detection limit. The RNA‐seq yielded for 50, 20, and 10 ng RNA input were 1.07 M, 350.5 K, and 181.2 K of read counts, respectively (Figure 2B). It is also noteworthy that we acquired more read counts and bases from the 10 ng input library using Im‐cpl protocol than the 100 ng input library using Sol‐RT protocol.

### 2.5 Phylotranscriptomic Identification of Foodborne Pathogens in a Low‐Input Cocktail Community Standard

The Im‐cpl protocol for low‐input RNA was further validated using a cocktail community standard composed of *Escherichia coli* O157:H7, *Salmonella Enteritidis*, and *L. monocytogenes* transcriptome (**Table** **2**). The cocktail included both Gram‐positive and Gram‐negative bacteria with different input levels (Table 2). A total of 50 ng of cocktail RNA was used as the input, and Notably, *L. monocytogenes* had the lowest RNA input of 1 ng. For comparison of the Im‐cpl and standard Sol‐RT methods for RNA quantification, each community RNA cocktail was mixed with Lexogen's synthetic spike‐in transcripts. The Im‐cpl protocol was successful in generating a high‐yield RNA library from a mixture of Gram‐positive and Gram‐negative bacteria, with a recovery rate of 100% while the Sol‐RT protocol exhibited an undetectable yield in the final library prepared from the same amount of the RNA cocktail. Phylotranscriptomic analysis reveals that a similar total yield of 152 and 121 K reads were obtained using the Im‐cpl and Sol‐RT protocols, respectively. However, the Im‐cpl method showed a lower failed reads rate (10.8%) and displayed higher fidelity with a greater mean read length (537 nt), compared to the Sol‐RT method with a higher failed reads rate (30.8%) and a much shorter mean read length (129 nt). MG‐RAST also successfully identified all three pathogens. The results shown in Table 2 show a satisfactory identification of the RNA from all three organisms using Im‐cpl protocol by recovering 12 164, 4158, and 2036 reads for *E. coli*, *S. Enteritidis*, and *L. monocytogenes*, respectively, considerably higher than the Sol‐RT results, recovering 158, 32, 10 reads, respectively. The RNA‐seq data analysis of the spike‐in transcript standards is described later.

**Table 2:** Bioinformatics analysis of the community standard sample using Im‐cpl protocol

Library preparation protocol		Im‐cpl	Sol‐RT
Community standard sample	Total input [ng]	50	
	*E. coli* O157:H7	39 (78%)	
	*Salmonella Enteritidis*	10 (20%)	
	*Listeria monocytogenes*	1 (2%)	
Total yield [reads]		151 877	121 037
Failed reads for analysis		16 331 (10.8%)	37 228 (30.8%)
Mean read length [nt]		537	129
Bioinformatics pipelines (MG‐RAST*)	*Escherichia*	12 164 (55.7%)	158 (69.0%)
	*Salmonella*	4158 (19.0%)	32 (14.0%)
	*Listeria*	2036 (9.3%)	10 (4.4%)

### 2.6 Poly(A) Tail Length Analysis

**Figure 3:**
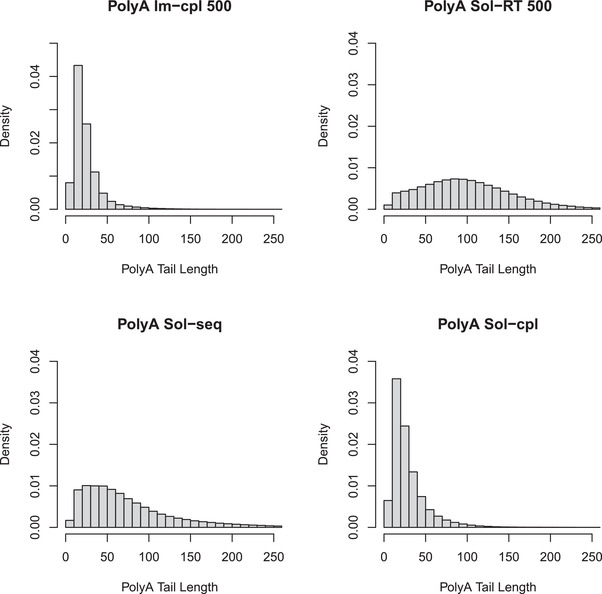
Poly(A) length analysis of Im‐cpl and Sol‐RT. Normalized 3′ poly(A) length subgroups were generated using the datasets produced with 500 ng *L. monocytogenes* RNA by the four protocols, depicted in Figure 2, with 10 nt bin size. The mean and median poly(A) lengths of different datasets are shown in Table S2 of the Supporting Information.

To validate the effect of enzymatic processing strategies on 3′ poly(A) tail length, we analyzed the 500 ng input libraries of four different protocols (**Figure** **3**). The data show that Im‐PAP and Im‐T4 DNA Ligase are capable of catalyzing the poly(A) tailing and adaptor ligation of a heterogeneous RNA population. As expected, the coupled reaction methods, with a mean length of 25 nt for Im‐cpl and 29 nt for Sol‐cpl, displayed substantially shorter poly(A) tail lengths than the sequential reaction protocol (Sol‐seq) and the Sol‐RT protocol. The two sequential reaction methods exhibited significantly longer poly(A) tails, possessing a mean 3′ poly(A) length of 75 nt for Sol‐seq and 101 nt for Sol‐RT. Poly(A) tails were more monolithic in coupled reactions than sequential reactions (Table S1, Supporting Information). Thus, the choice of enzymatic processing approach, i.e., coupled reactions versus sequential reactions, plays a major role in determining the length and range of poly(A) tails. The sequencing yield results show that the 3′ poly(A) tail lengths observed with the Im‐cpl and Sol‐cpl protocols are sufficient for the capture and ligation with the RTA adaptor.

### 2.7 Library Quality and Reads Characteristics

To more thoroughly assess the capability and quality differences of the library preparation protocols presented in this study, we performed analyses to compare various quality metrics of RNA sequencing datasets generated. The RNA reads were mapped against the reference genome of *L. monocytogenes* EGD‐e. All protocols displayed satisfactory mapping results, generating a mapped rate of ≈99% before rRNA sequences were removed. The results further demonstrated a satisfactory RNA mapping rate of 84% from the Im‐cpl protocol, a significant improvement from 79% from the Sol‐RT protocol, after rRNA sequences were removed (Figure S9, Supporting Information). Results also show that a mean of 10.1 to 10.3, a median of 10.2 to 10.5 of read quality were generated from all five protocols. After rRNA removal, a slight decrease of mean and median was also observed from all five protocols, suggesting the read quality was not compromised by using the Im‐cpl method for 500 ng RNA‐seq (Table 1). When the Im‐cpl protocol was used for low‐input library preparations, the mapped rates were still greater than 99% for 100 and 50 ng input and were greater than 98% with even lower RNA input (20 and 10 ng) validated. The results also demonstrate that read quality generated from low RNA input was not significantly different from that generated using 500 ng (Table S2, Supporting Information).

### 2.8 Comparative Transcriptome Analysis

**Figure 4:**
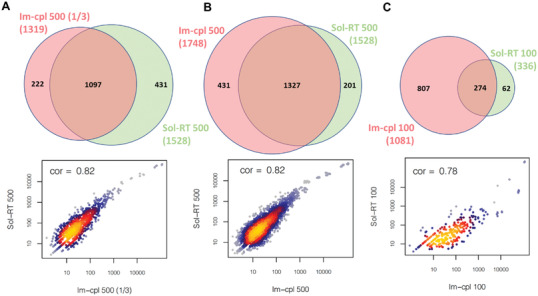
Venn Diagrams and transcripts per million (TPM) Pearson correlations of RNA‐seq data from the Im‐cpl and Sol‐RT protocols and different *L. monocytogenes* RNA input amounts. A) Im‐cpl 500 (1/3) (single flow cell run of one‐third of the RNA library) and Sol‐RT 500. B) Im‐cpl 500 (the entire library run on three flow cells) and Sol‐RT 500. C) Im‐cpl 100 and Sol‐RT 100.

In addition to the reads and bases generated from the protocol using Im‐cpl and Sol‐RT for both 500 and 100 ng low‐input of RNA, we also sought to evaluate whether our new approach can provide better biological insights into the transcriptome. Among all the transcriptome analyses, the comparison of two replicates from each of the protocols (i.e., Im‐cpl 500, Sol‐RT 500, Im‐cpl 100, and Sol‐RT 100) was first performed to evaluate the variability of each library preparation protocol. The average genes identified by the two replicates from each of the Im‐cpl 500, Sol‐RT 500, Im‐cpl 100, and Sol‐RT 100 protocols are 1309, 1504, 954, and 442 genes, respectively. While the replicates of the Im‐cpl methods produced consistent results in both gene content and transcript quantities, there is a larger variation between the Sol‐RT replicates with 100 ng of RNA input (Table S3 and Figure S10, Supporting Information). After comparison of two replicates from each protocol, the replicate with more reads generated by RNA‐seq from each protocol was used for a cross‐protocol comparison. It is also worthy to notice that only one‐third of the Im‐cpl RNA library prepared from 500 ng input [Im‐cpl 500 (1/3)] provided an equivalent amount of material as the entire library of the other protocols and was used for sequencing on one flow cell. The Im‐cpl 500 (1/3) library identified a comparable number of genes (1319) as the Sol‐RT 500 library (1528), and their common genes showed a good correlation of 0.82 (Pearson's correlation) of normalized read counts (**Figure** **4**). To capture all the information from the whole Imp‐cpl 500 library, we also performed another set of experiments to have the entire RNA library prepared from 500 ng RNA sequenced using three flow cells (Figure S11, Supporting Information). The data from all the three flow cells were combined (Im‐cpl 500), and 220 more genes were identified (1748 genes in total) than the Sol‐RT 500 library of 1528 genes (Figure 4). This further demonstrated that the Im‐cpl protocol could generate more data and information than the Sol‐RT protocol when the entire RNA library material was used for sequencing from the same amount of RNA input.

We further analyzed the read lengths from the libraries prepared with the Im‐cpl and Sol‐RT protocols using both 500 ng and 100 ng low RNA input amount. The results shown in Figure S12 of the Supporting Information demonstrated a longer mean read length from Sol‐RT than Im‐cpl with 500 ng RNA input, demonstrating Sol‐RT performed better in long RNA retaining and sequencing. This difference, however, was not significant when rRNA sequences were removed. For lower input amount (100 ng), no significant mean read length change was observed before and after rRNA removal. It is also noteworthy to note that Im‐cpl revealed longer mean and medium read length compared to Sol‐RT when lower RNA input amount was used, which corroborated the results observed when 50 ng of a community standard was used for RNA‐seq, in which mean read lengths are 537 and 129 nt for Im‐cpl and Sol‐RT, respectively.

Comparison of the low‐input RNA‐seq libraries showed that when 100 ng of RNA was applied as an example of low‐input RNA, the Im‐cpl protocol (Im‐cpl 100) identified twice more genes (1081 genes) than the Sol‐RT protocol (Sol‐RT 100), which identified only 336 genes (Figure 4). The data clearly demonstrate that although the Im‐cpl protocol performed similarity to Sol‐RT for a single flow cell run when 500 ng of RNA were applied, it exhibits better performance in generating higher data output and identifying more expressed genes for low‐input RNA‐seq application. The protocol‐wise comparison also revealed that RNA input amount does not have a significant effect on the transcriptome profiling results for the Im‐cpl protocol as the identified genes using the Im‐cpl protocol decreased slightly from 1319 to 1081 as RNA input decreased from 500 to 100 ng, and the correlation of transcript counts between the two (Im‐cpl 500 and Im‐cpl 100) is 0.76 (Figure S13, Supporting Information). By contrast, the performance of the Sol‐RT protocol decreased drastically from identifying 1528 genes to only 336 as RNA input amount decreased from 500 to 100 ng (Figure S13, Supporting Information). These results further demonstrate that the Im‐cpl protocol is less affected by the lack of RNA input as opposed to Sol‐RT, and therefore, the Im‐cpl protocol appears to be a more robust method than the Sol‐RT protocol for studying various samples of a wide range of quantities.

**Figure 5:**
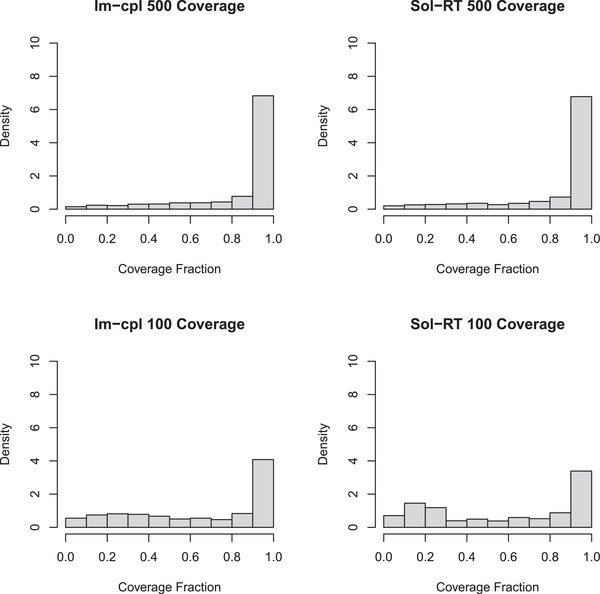
The coverage of genes identified from various protocols with different RNA input amounts. A) Gene coverage from the entire Im‐cpl 500 library (three sequencing run datasets combined) from a single 500 ng input library. B) Gene coverage from a Sol‐RT 500 ng library (Sol‐RT 500). C) Gene coverage from Im‐cpl protocol with 100 ng of RNA input (Im‐cpl 100). D) Gene coverage from Sol‐RT protocol with 100 ng of RNA input (Sol‐RT 100).

We also examined and compared the sequencing coverage of captured genes by the two different methods (**Figure** **5**). The per‐gene average coverage achieved by the Im‐cpl 500 (entire library) method is 85.8%, similar to the coverage (84.9%) achieved from the Sol‐RT 500 library. And both Im‐cpl 500 and Sol‐RT 500 libraries produced a median per‐gene coverage of 100%, indicating more than half of the genes were fully sequenced using either method. For the 100 ng RNA input libraries, a higher mean (66.7%) and a higher median (78.5%) per‐gene coverage were achieved from the Im‐cpl protocol than the mean (60.2%) and median (66.5%) obtained from the Sol‐RT protocol (**Table** **3**).

**Table 3:** Sequencing coverage of common genes and unique genes of the Im‐cpl using the entire library and the Sol‐RT with different RNA input amounts

Protocols	Mean [%]	Median [%]	Number of genes
Im‐cpl 500	85.8	100	1319
Sol‐RT 500	84.9	100	1528
Im‐cpl 100	66.7	78.6	1081
Sol‐RT 100	60.2	66.5	336

**Figure 6:**
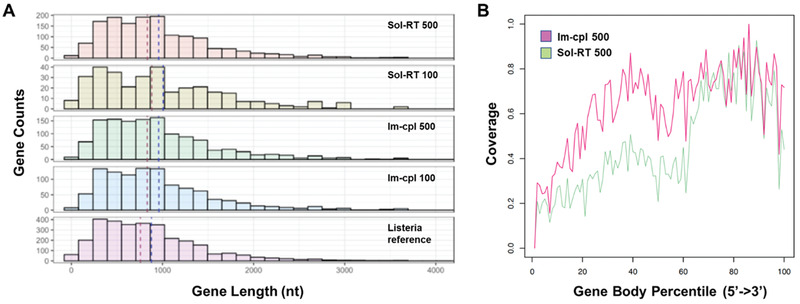
Distribution of gene counts at different gene lengths and sequencing coverage of gene body from 5′ to 3′ by Im‐cpl and Sol‐RT protocols with 500 ng RNA input. A) Distribution of gene counts at different gene lengths of the common genes and the unique genes of the Im‐cpl and Sol‐RT methods in comparison to the distribution of the *L. monocytogenes* reference genome. B) Sequencing coverage of gene body from 5′ to 3′ by Im‐cpl and Sol‐RT protocols with 500 ng RNA input.

Comparison of coverage of *L. monocytogenes* transcriptome also showed that both Im‐cpl and Sol‐RT libraries produced similar gene coverage distribution and resemble *L. monocytogenes* transcriptome when 500 ng of RNA input amount was used for library preparation. On the other hand, the Im‐cpl method offered better transcriptome coverage than the Sol‐RT protocol for 100 ng low RNA input compared with the reference (**Figure** **6**). Furthermore, data from Figure 6 also demonstrated better 5′ end sequence coverage by the Im‐cpl method compared to the Sol‐RT protocol. These results combined demonstrate that the Im‐cpl method offers comparable performance with the Sol‐RT method in transcriptome profiling when applied to 500 ng input RNA. Importantly, when a low amount of input material (e.g., 100 ng) is used, Im‐cpl protocol outperformed Sol‐RT in providing a more complete and less biased set of transcripts with better gene coverage and quantification accuracy. In addition to the comparison of Im‐cpl with Sol‐RT, we also compared our Im‐cp method with the Sol‐seq protocol using 500 ng input RNA in case researchers would like to perform RT‐free experiments for direct RNA‐seq. Our results showed that the Im‐cpl protocol also outperformed Sol‐seq in providing higher read yield, a more complete and less biased transcriptome profile (Figure S14, Supporting Information).

Finally, the use of Lexogen's synthetic spike‐in transcripts in RNA‐seq analysis of the community cocktail sample (Table 3) allowed us to further compare the new Im‐cpl method and the standard Sol‐RT method in RNA quantification. We examined external RNA controls consortium (ERCCs) and long Spike‐in RNA variants (SIRVs) and found that for low‐input RNA amount, the Im‐cpl protocol produced significantly higher coverage of ERCCs and long SIRVs (Table S4, Supporting Information). Notably, the Im‐cpl method recovered all 15 long SIRV transcripts, whereas the standard Sol‐RT method detected only 5 long SIRV species (Figure S15, Supporting Information). This result is consistent with the observation that significantly higher reads were recovered for the bacterial pathogens present in the community sample (Table 3).

## 3 Discussion

In this work, we have developed a novel method for RNA library preparation for non‐polyadenylated RNAs in direct RNA‐seq analysis using ONTs third‐generation sequencing platform using bacterial pathogen *L. monocytogenes* as a model. This solid‐phase catalysis approach employs immobilized enzymes to circumvent the use of bead‐based purification that causes significant sample loss. In conjunction with a coupled enzymatic reaction scheme, this new workflow uses a one‐pot formulation to produce a sequencing‐ready library for direct loading onto a MinION flow cell for a sequencing run. Owing to its ability to avoid sample loss, our new method significantly lowers the input threshold for direct metatranscriptomic RNA‐seq. Compared to the current method, the method presented exhibits an improved transcriptome profiling, including the identification of a larger number of expressed genes per 500 ng input amount and higher 5′ region sequence coverage. In addition, we demonstrate the identification of considerably more transcripts with low input amounts and, in particular, in a mock community pathogen cocktail.

Whole transcriptome sequencing aims to acquire a global picture of all RNA transcripts of an organism. For example, researchers can take advantage of the detection of RNA biomarkers to measure bacterial viability because of the significantly shorter half‐life of RNA molecules than DNA molecules.^[^ ^16^ ^]^ Our previous study indicates that direct RNA‐seq has the potential for multiplex identification of viable bacterial pathogens without the need to customize for individual bacteria; however, the requirement of large amounts of input RNA has limited its use in sensitive surveillance.^[^ ^12^ ^]^ Conceivably, the adoption of various techniques and practices can greatly influence the research outcome due to the presence of artifacts and limitations. Since its initial development, SPRI beads technology has been utilized in a variety of nucleic acid preparation chemistries, including enrichment, size selection, and removal of enzymes and other reactants.^[^ ^17^ ^]^ This process, however, is prone to sample loss and artifacts in handling and presents a major challenge for the use of native RNA‐seq for whole transcriptome analysis. Bead‐based purification in the current soluble enzyme protocol may cause certain selection effects by preferentially binding and eluting transcripts of different sizes and compositions or simply a loss of low expression gene transcripts. Thus, despite broad efforts in improving bioinformatics tools to reduce bias introduced during data processing, minimizing the bias that occurs during library preparation is the first and most critical step to avoid erroneous interpretation of the data and their biological relevance.

In this work, we have established a new strategy for constructing the RNA library that eliminates widely used bead purification during library preparation, thereby enabling direct RNA‐seq without significant sample loss. We have found that this approach significantly lowers the threshold for direct metatranscriptomic RNA‐seq. Direct RNA sequencing was achieved by using as little as 10 ng of input RNA, a small fraction of the 500 ng input required by the current ONT protocol. We further demonstrated successful sequencing and mapping from a pathogen cocktail possessing 1 ng of *L. monocytogenes* RNA input. Our new method can improve the identification and quantification of RNA for low input amount is evident when the spike‐in transcripts were analyzed and compared to the current soluble enzyme method.

The new strategy was designed based on solid‐phase enzyme catalysis by covalent conjugation of two relevant enzymes to magnetic microbeads. In conjunction with a coupled enzymatic processing scheme, we created the one‐pot formulation for fast and robust RNA library preparation workflow, potentially enabling ONT's long‐read sequencing for native RNA molecules from rare and difficult‐to‐obtain, such as clinical specimens or environmental samples. Our strategy has consistently resulted in higher sequencing yields with satisfactory qualities and more complete transcriptome profiles of *L. monocytogenes* from 500 ng to very low amounts (e.g., 10 ng) of input RNA. Our solid‐phase catalysis strategy, for the first time, allows for both enzymatic processing and enzyme removal from the final RNA library and thus enables the preparation of sequencing‐ready libraries without bead‐based purification, a widely adopted process in many NGS workflows. The coupled reaction protocol using immobilized enzymes avoided bead‐based clean‐up, resulting in substantial reduction of sample loss and identification of more transcripts for *L. monocytogenes*, with both 100 and 500 ng input amounts and 1 ng *L. monocytogenes* input in the community sample. The higher transcript identification rate is consistent with better recovery of the spike‐in RNA controls. The data suggest that our new methodology may help to capture a more complete transcriptome that may offer insight into the nature and expression of the transcriptome. This finding is important for the choice of reliable library preparation for RNA‐seq studies because accurate measurement of RNA transcript sequences and their copy numbers is a foundation for understanding dynamic transcript expression^[^ ^4^, ^18^ ^]^ (8,39). Therefore, the method presented may potentially reduce false interpretation of transcriptomic data, as unbiased RNA‐seq data are critical for accurate quantification of gene expression, differential gene expression, and post‐transcription modifications.^[^ ^19^ ^]^ Further study is required to determine whether this method is applicable to a low‐input RNA‐seq analysis of various sources of rare and difficult‐to‐purify samples, including clinical human specimens. It is also of interest to examine if our new method can facilitate RNA‐seq analysis of other non‐polyadenylated RNAs such as long noncoding RNAs that play major roles in cancer.^[^ ^14^, ^20^ ^]^

Our data demonstrate that the enzyme immobilization, in conjunction with the coupled enzymatic reaction strategy, has generated a fast and streamlined workflow for ONT RNA library preparation. The current protocol using soluble enzymes requires 2–4 h to complete library preparation owing to its multiple sequential reactions, with each reaction performed in a different buffer, and up to three bead‐based clean‐up steps (each after poly(A) tailing, RTA ligation for RT, if any, and RMX ligation) (Figure 1). The new protocol has successfully eliminated bead‐based purification with less hands‐on time, and all the reaction components can be added together for 30 min one‐pot processing of RNA library in a single buffer system (Figure 1). The high data throughput is achieved by retaining the sequencing capability of nanopore sensors with a lower fouling rate than the current protocol using soluble enzymes. A number of factors may contribute to nanopore fouling. The presence of the protein molecules, i.e., poly(A) polymerase and T4 DNA ligase, can presumably cause clogging of the nanopores, thereby impairing their sequencing function. In this study, the other reagents present in the library medium do not appear to have an adverse effect on the Nanopore sequencing function because the immobilized enzyme protocols caused a lower nanopore fouling rate and produced more reads than the soluble enzymes protocols with or without bead purification. It is conceivable that direct loading of a prepared library to a flow cell without bead purification requires input RNA free of substantial amounts of factors that adversely affect Nanopore sequencing capability.

Our data confirmed that the Sol‐RT protocol with the incorporation of reverse transcription step can drastically increase the throughput of direct RNA‐seq compared to the Sol‐seq and Sol‐cpl protocols, as previously described.^[^ ^2^ ^]^ The Sol‐RT protocol, however, demands longer library preparation time than the Im‐cpl protocol significantly. The Im‐cpl method utilizes a much shorter yet highly efficient workflow. It generates a comparable throughput as the Sol‐RT protocol with 500 ng input but significantly higher yields and more accurate transcriptome profiling for low RNA inputs. The Sol‐RT library generated similar read output with a slightly higher number of genes identified compared to a flow cell run from one‐third of a typical Im‐cpl library; however, the latter can be entirely sequenced by multiple flow cell runs, resulting in both higher read output and identification of higher number of expressed genes. Furthermore, this highly streamlined Im‐cpl protocol is better suitable for automated library preparation and sequencing workflow.

It is noteworthy that both coupled enzymatic reaction protocols, Sol‐cpl and Im‐cpl, yielded significantly more reads than the sequential reaction protocol (Sol‐seq), albeit they have the same total 30 min reaction time. Our preliminary study indicates that *E. coli* poly(A) polymerase has low processivity and incorporates ≈1–2 nucleotide(s) per substrate binding event. Following the coupled reaction scheme, both PAP and ligase can potentially function during the entire 30 min incubation time, increasing the overall efficiency of adaptor ligation to the RNAs in a library. An RNA molecule with a sufficient length of 3′ poly(A) tail can anneal to an RTA possessing a single‐stranded sequence of 10 bases of dT at 3′ end. Consequently, adaptor ligation would deplete the pool of the unligated RNA molecules over time, thereby increasing the effective concentration of poly(A) polymerase to process the remaining RNA substrates. Indeed, our poly(A) tail length analysis revealed significantly shorter poly(A) tail length profiles when the couple reaction scheme was performed with either soluble or immobilized enzymes. Slightly longer poly(A) length associated with soluble enzyme treatment is likely due to faster kinetic property and/or higher soluble enzyme activity than the immobilized form. It has yet to be determined whether poly(A) length can influence Nanopore sequencing performance. On the other hand, the fact that the Sol‐RT method produced the longest mean 3′ poly(A) tail and the high read output indicates that 3′ poly(A) tail length is unlikely a major factor. Moreover, the coupled reaction protocol allows for ligase to function for the entire 30 min period compared to 10 min ligation with the sequential reaction protocol.

Solid‐phase catalysis based on immobilized enzymes can bring forth many unique benefits to the molecular workflows for Nanopore RNA‐seq, including enzyme removal without heat inactivation and bead‐based purification, thereby facilitating many enzyme‐based applications. Successful implementation of enzyme immobilization technology requires innovative solutions to overcome potential challenges such as lower specific activity, higher costs, and additional equipment needs. Our previous study indicates that immobilization of enzymes can result in lower enzymatic activity in comparison to their soluble counterpart, and higher activity of the immobilized form can be achieved by surface modification of the magnetic microbeads.^[^ ^21^ ^]^ This approach has significantly enhanced the enzymatic activity of *E. coli* poly(A) polymerase. In addition, immobilized DNA ligase displayed proficient efficiency in adaptor ligation despite the fact that fewer enzyme units were used in place of highly concentrated soluble T4 DNA Ligase. Therefore, our work presented has demonstrated the utility of immobilized enzymes and can potentially contribute to the establishment of a highly efficient and less biased RNA library method. With high‐throughput sequencing already being applied in clinical contexts, there is a pressing need for the automation of multireaction workflows.^[^ ^22^ ^]^ With this new tool, we can explore new possibilities for a highly integrated system for fully automated library preparation, sequencing, and analysis in various research and clinical applications.

## 4 Experimental Section

### Materials

Ni‐NTA Agarose was purchased from Qiagen (Cat #. 30230) for protein purification. RNA Clean XP beads were obtained from Beckman Coulter, Inc. (Cat #. A63987) for bead‐based purification for RNA library preparation. *E. coli* O157:H7 (ATCC 43895), *Salmonella enterica* serovar *Enteritidis* (ATCC 13076), and *Listeria monocytogenes* (ATCC 19115) were acquired from the American Type Culture Collection (Manassas, VA). Monarch Total RNA Miniprep Kit from New England Biolabs, Inc. (NEB, T2010S) was used for total RNA extraction. Magnetic beads were purchased from General Electric and modified for enzyme immobilization.^[^ ^21^ ^]^ SuperScript III reverse transcriptase (Ref #. 18080–093) was purchased from Invitrogen by Thermo Fisher Scientific. Spike‐in RNA variant controls (SIRV‐Set 4) were purchased from Lexogen. All other soluble enzymes were obtained from NEB. 20× phosphate buffer saline (#9808) was purchased from Cell Signaling Technologies, MA, USA. The chemical reagents are in analytical grade.

### Protein Purification

The coding sequence of *E. coli* PAP was subcloned into pSNAP_f_‐tag (T7) (NEB) using NEBuilder (NEB). The resulting plasmid pPASH was used to express and purify a fusion protein (termed PASH) comprised of *E. coli* PAP, SNAP‐tag, and six‐histidine tag following the protocol described previously.^[^ ^15^, ^21^ ^]^ *E. coli* T7 Express (NEB, C2566) transformed with pPASH was inoculated to 5 mL of LB/ampicillin medium and cultured at 37 °C for 4 h. 1 mL of subculture was further inoculated to 1 L of LB/ampicillin medium and incubated at 37 °C for about 3–4 h until OD_600_ reached 0.6–0.7. Then 3 mL of 100 × 10^−3^ m isopropyl *β*‐d‐1‐thiogalactopyranoside was added to the medium (with a working concentration of 0.3 × 10^−3^ m) to allow the expression of SNAP‐tagged poly(A) polymerase recombinant protein (PASH). The expression was carried out at 16 °C overnight. The culture was then harvested and centrifuged to discard the supernatant. The pellets were then lysed using a sonicator on ice and centrifuged for crude extract. The clarified cell lysate was loaded onto Ni‐NTA resin, followed by extensive washing with wash buffer (50 × 10^−3^ m Tris buffer, pH 8, 20 × 10^−3^ m imidazole, and 0.3 m NaCl). The fusion protein was then eluted using elution buffer (50 × 10^−3^ m Tris buffer, pH 8, 250 × 10^−3^ m imidazole, and 0.3 m NaCl). The eluted protein was dialyzed against Diluent C buffer No BSA (NEB) overnight and stored for future experiments. The concentration of enzymes was determined using the Bradford assay. The kinetic studies were carried out as described for NEB poly(A) polymerase.

### Enzyme Immobilization

SNAP‐tagged T4 DNA Ligase immobilized to O^6^‐benzylguanine (BG) modified magnetic beads were characterized to catalyze ligation of various substrates (Figure S2, Supporting Information).^[^ ^15^ ^]^ PASH was immobilized to magnetic beads modified with BG or BG‐PEG_4_ ligand with or without PEG_750_ coating as previously described,^[^ ^21^ ^]^ to generate three immobilized forms, BG‐PASH, BG‐PASH (PEG_750_), BG‐PEG_4_‐PASH (PEG_750_) (Figure S3, Supporting Information). 100 µL of BG beads slurry (25%) was washed with 250 µL buffer solution (1 × PBS, 1 × 10^−3^ m DTT, and 300 × 10^−3^ m NaCl) five times. Next, 25 µg PASH protein was dissolved to 125 µL of the buffer solution and further loaded to the washed beads. The mixture of enzyme and beads was incubated overnight at 4 °C. The beads immobilized with PASH were washed using the same buffer 8 times to remove the excessive enzymes. Diluent C buffer with no BSA (NEB) was used to resuspend the beads and for storage at −20 °C. Immobilized poly(A) polymerase with different modification strategies (i.e., BG‐PASH, BG‐PASH (PEG_750_), and BG‐PEG_4_‐PASH (PEG_750_)) was evaluated using capillary electrophoresis, and BG‐PASH (PEG_750_) proved to be the immobilized form with the highest activity, which was stored and used for the following experiments presented (Figure S3, Supporting Information). The poly(A) tailing function of the immobilized poly(A) polymerase was also demonstrated through the poly(A) length analysis (Figure 3).

### RNA Extraction

*L. monocytogenes* was cultured using Brain Heart Infusion (BHI) broth at 37 °C for 22 h. Total RNA extract of *L. monocytogenes* was performed according to the previous report.^[^ ^12^ ^]^ Particularly, 1 mL of the culture was centrifuged at 16 000 × *g* for 2 min to harvest the cell pellet. 250 µL of 3 mg mL^−1^ lysozyme diluted in TE buffer was added to suspend the cell pellet followed by incubation at 37 °C for 1 h. Total RNA was extracted using the Monarch Total RNA Miniprep Kit (NEB, T2010). Specifically, 40 µL of RNA with DNA contamination sample was eluted in nuclease‐free water. Then, 20 µL of DNase I (NEB, cat. # T2004‐1, 20 U), and 20 µL of DNase I reaction buffer were added to the eluted sample and incubated at 37 °C for 30 min for DNA digestion. Finally, RNA Priming Buffer and Wash Buffer were used for RNA purification. The RNA extract was quantified by high sensitivity Qubit Assay and Nanodrop, respectively, and stored at −80 °C. A similar protocol was used for preparing the community standard, which consists of *E. coli* O157:H7, *S. Enteritidis*, and *L. monocytogenes*. Particularly, a single colony of each strain was inoculated in 10 mL of BHI medium, respectively, followed by incubation at 37 °C for 24 h. 2 mL of each overnight culture was used for total RNA extraction using NEB Monarch Total RNA Miniprep Kit.^[^ ^12^ ^]^

### RNA Library Preparation

Sequential reaction protocols start RNA library preparation with poly(A) tailing using poly(A) tailing, followed by ligation of poly(A) tailed RNA with adaptors using T4 DNA ligase. These two steps can be performed simultaneously in one‐pot (coupled reaction protocols) using soluble or immobilized enzymes.

All libraries with 500 ng of *L. monocytogenes* total RNA were prepared using various protocols (Figure 1). For the sequential protocol using soluble enzymes (Sol‐seq), the following reagents were mixed: 8 µL of quick ligation buffer, 1.2 µL of 5 m NaCl solution, 0.5 µL of poly(A) polymerase (NEB, M0276), and 500 ng of *L. monocytogenes* total RNA. The mixture was supplemented with nuclease‐free water to 30 µL in a 0.2 mL thin‐walled PCR tube. The mixture was incubated at 37 °C for 20 min for poly(A) tailing. 1 µL of RTA, 6 µL of RMX, and 3 µL of T4 DNA ligase were then added to the poly(A) tailed RNA sample to make a final volume of 40 µL. The mixture was incubated at 25°C for 10 min to allow the ligation of the adaptors to the poly(A) tailed RNA samples. For the coupled reaction protocol using soluble enzymes (Sol‐cpl), all components used for the Sol‐seq reaction protocols were mixed at once, as a one‐pot reaction. 40 µL of the mixture was subjected to incubation at 37 °C for 20 min, followed by 25 °C for 10 min. The libraries prepared by Sol‐seq and Sol‐cpl were purified with RNAClean XP beads as follows: 40 µL of resuspended RNAClean XP Beads was added to the 40 µL of adapter‐ligated RNA and mixed by pipetting. The reaction mixture was incubated on a Hula mixer (rotator mixer) at room temperature for 5 min, and the RNA sample was pelleted on a magnet. The supernatant was pipetted off, and 150 µL of the Wash Buffer was added to the beads and resuspended for washing. The beads were pelleted, and the supernatant was pipetted off. The previous step was repeated. The tube was removed from the magnetic rack, and the pellet was resuspended in 21 µL of Elution Buffer. The mixture was incubated at room temperature for 10 min to allow the elution of RNA. Beads were pelleted on a magnet until the eluate was clear and colorless. 21 µL of the eluate was collected and retained into a clean 1.5 mL Eppendorf DNA LoBind tube. RNA concentration was measured using high sensitivity Qubit Assay Kit, and the final yield and recovery rate were determined. In addition, a pair of libraries were prepared according to the Sol‐seq protocol using soluble enzymes without bead‐based purification [Sol (‐BP)]. For the *L. monocytogenes* RNA libraries of 500 ng input prepared by the coupled method using immobilized enzymes (Im‐cpl protocol), 2.5 µL of immobilized poly(A) polymerase (BG‐PASH (PEG_750_)) and 3 µL of immobilized T4 DNA ligase was applied to replace their soluble counterparts. Both immobilized poly(A) polymerase and immobilized T4 DNA ligase were removed after the one‐pot reaction from Im‐cpl mixture. Due to the usage of immobilized enzymes, bead‐based purification was not applied for the Im‐cpl method.

*L. monocytogenes* RNA libraries with low‐inputs of 100, 50, 20, and 10 ng were prepared using the Im‐cpl protocol by mixing the following components in a 0.2 mL thin‐walled PCR tube: 3 µL of quick ligation buffer, 0.45 µL of 5 m NaCl solution, 1.5 µL of immobilized poly(A) polymerase, and *L. monocytogenes* total RNA (e.g., 100, 50, 20, and 10 ng, respectively). 0.5 µL of RTA, 3.0 µL of RNA RMX and 1.5 µL of Im‐ligase and nuclease‐free water were added to make a final volume of 15 µL. The reaction was incubated at 37 °C for 20 min and 25 °C for 10 min. The immobilized poly(A) polymerase and immobilized T4 DNA ligase beads were removed from the reaction mixture by placing the tube on the magnetic rack.

*L. monocytogenes* RNA libraries were also performed using soluble enzymes with a reverse transcription step (Sol‐RT) as recommended by ONT (Figure 1) for both 500 ng (Sol‐RT 500) and 100 ng (Sol‐RT 100) input RNA‐seq by mixing the following components: 3 µL of quick ligation buffer, 0.45 µL of 5 m NaCl solution, 0.5 µL of soluble poly(A) polymerase (NEB), and different inputs of RNA, 500 ng (Sol‐RT 500) and 100 ng (Sol‐RT 100), respectively. The mixture was supplemented with nuclease‐free water to 12.5 µL and incubated at 37 °C for 20 min for poly(A) tailing. Next, 1 µL RTA and 1.5 µL T4 DNA ligase were added to a final volume of 15 µL. The mixture was then incubated at 25 °C for 10 min to allow RTA ligation. Then, 9 µL nuclease‐free water, 2 µL 10 × 10^−3^ m dNTP, 8 µL first strand buffer, 4 µL 0.1 m DTT, and 2 µL SuperScript III reverse transcriptase were added to a final volume of 40 µL. The reverse transcription reaction was performed at 50 °C for 50 min followed by 70 °C for 10 min. RNA samples were purified with RNAClean XP beads after treatment with soluble enzymes. 72 µL of resuspended RNAClean XP Beads was mixed with the 40 µL of adapter‐ligated RNA by pipetting, followed by incubation on a Hula mixer (rotator mixer) at room temperature for 5 min. The bead fraction, pelleted on a magnet, was washed with 150 µL of fresh prepared 70% ethanol. RNA was eluted in 20 µL of nuclease‐free water by incubation at room temperature for 5 min. The eluted RNA was then mixed with 8 µL quick ligation buffer, 6 µL RMX adaptor, 3 µL nuclease‐free water, and 3 µL T4 DNA ligase, and the mixture was incubated at 25 °C for 10 min for RMX ligation. The final bead‐based purification was carried out as described above for Sol‐seq and Sol‐cpl. RNA yields were determined by high sensitivity RNA Qubit assays.

A pair of community standard libraries were prepared by the low‐input Im‐cpl protocol and Sol‐RT protocol, respectively. Each library is composed of 39 ng of *E. coli* O157:H7, 10 ng of *S*. *Enteritidis*, 1 ng of *L. monocytogenes* RNAs, and 1 ng of Spike‐in RNA variant control (SIRV‐Set 4) mixture provided by Lexogen.

### Nanopore Direct RNA sequencing

Direct RNA sequencing of the prepared libraries was performed on ONTs GridION using R9.4 flowcells. Each sequencing run was performed until less than ten active nanopores were available for RNA seq, or 72 h of sequencing running was achieved, which comes earlier. For the RNA libraries prepared using soluble enzymes (Sol‐seq and Sol‐cpl) and the protocols with reverse transcription (Sol‐RT 500 and Sol‐RT 100), 20 µL of each sample recovered from bead purification was mixed with 17.5 µL of nuclease‐free water to 37.5 µL. The mixture was further mixed with 37.5 µL of RNA running buffer (RRB) to a final volume of 75 µL for loading into a flow cell. For the libraries prepared by a coupled reaction approach using immobilized enzymes with one‐third of the library being loaded [Im‐cpl 500 (1/3)], or by soluble enzymes without bead purification [Sol (‐BP)], an equivalent amount of RNA comparable to that of the sequential approach using a Sol‐seq protocol was used for a sequencing run; the volume was first brought up to 37.5 µL by addition of nuclease‐free water followed by mixing with 37.5 µL of RRB to a final volume of 75 µL. The entire Im‐cpl library with 500 ng input (Im‐cpl 500) was subjected to three flow cell runs with each flow cell loaded with 13 µL of the library. For low‐input RNA libraries (Im‐cpl 100, 50, 20, and 10), 13 µL of each 15 µL prepared sample was mixed with 24.5 µL of nuclease‐free water to a final volume of 37.5 µL before the addition of 37.5 µL RRB for a sequencing run.

### Data Characterization and Low Input RNA‐seq

Raw sequencing reads were collected via a local base‐calling algorithm with MinKNOW, base‐called using Guppy, and aligned to the *L. monocytogenes* reference genome using Minimap2. All FastQ files of passed base‐called reads were collected combined for future analysis, and only reads present in the “pass” folder were used in subsequent analysis. Comparison across experiments of the read length and distribution, number of reads, and reference identity were performed using NanoComp.

### Community Sample Analysis

The FastQ files of passed reads of the community sample libraries were first extracted from the base‐called fast5 files and were analyzed via MG‐RAST for metatranscriptomic analysis and taxonomic identification.^[^ ^23^ ^]^ The passed reads were also aligned to the ribosomal DNA sequences of *L. monocytogenes, S. enterica*, *and E. coli* using minimap2.^[^ ^24^ ^]^ Subsequently, the unmapped non‐RNA reads were mapped to the composite reference genome of the three aforementioned organisms, and reads were differentiated from organisms using Samtools v1.9.

### SIRV Long Range and ERCC Standard Analysis

SIRV‐Set 4 (Cat. No. 141, Lexogen) contains the long SIRV with 15 long SIRV RNAs of 4–12 k and ERCC standard RNAs. The non‐RNA reads from the community sample libraries were mapped to the long SIRV standard sequences (SIRV_longSIRVs_multi‐fasta_200709a.fasta) and the ERCC standard RNA sequences (SIRVome_isoforms_ERCCs_longSIRVs_200709a.fasta). The mapped read counts were calculated using Samtools v1.9. Read counts and mapped read length were plotted in R studio 1.3.1073.

### Poly(A) Tail Analysis

For poly(A) tailing analysis, Oxford Nanopore raw reads were base called using guppy base caller (2020 Oxford Nanopore Technologies, Ltd). Each of the large datasets was divided into 20 smaller subsets and used the tailfindr v1.2 program to deduce poly(A) tail lengths of all of the base‐called reads.^[^ ^25^ ^]^ Statistical analysis of poly(A) tail size of different samples was conducted in R studio 1.3.1073.

### Alignment Length Analysis

The FastQ files were first extracted from the base‐called fast5 files and then aligned to the ribosomal DNA sequence of the *L. monocytogenes* genome using minimap2 and unmapped output reads.^[^ ^24^ ^]^ The non‐rRNA reads were then mapped to the *L. monocytogenes* reference genome (NC_003210), and only primary alignments were used for downstream analysis. The resulting alignment bam files were converted to bed files by bedtools v2.29.2,^[^ ^26^ ^]^ and the alignment sizes were computed from the bed files. Plotting of alignment size distributions of different samples was carried out in R studio 1.3.1073.^[^ ^27^ ^]^

### Gene Expression Analysis

The featureCount v2.0.1 was used to quantify reads that map to individual genes in the *L. monocytogenes* reference genome from the minimap2 alignments.^[^ ^28^ ^]^ Gene counts were normalized by gene lengths and the total number of aligned reads. Normalized read counts TPM were used for generating scatter plots and measuring linear correlation by Pearson correlation coefficiency between the datasets in R studio v1.3.1073. The raw gene count matrices of two technical replicates of the Im‐cpl and the Sol‐seq datasets were used for differential gene expression analysis using the Bioconductor package DESeq2 v3.12 in R Studio v1.3.1073.^[^ ^29^ ^]^

### Sequencing Coverage Analysis

The bedCoverage command of the bedtools (v2.29.2) was used to calculate sequencing coverage of individual genes based on the minimap2 alignments and the gene annotation of the *L. monocytogenes* reference genome (NC_003210) downloaded from NCBI. Gene body sequencing coverage was analyzed and visualized using the RSeQC package.^[^ ^30^ ^]^

### Statistical Analysis

For sequencing yields analysis, sequencing yields from Sol‐seq, Sol‐cpl, and Sol (‐BP) with 500 ng RNA input were repeated twice. Sol‐RT with 500 ng input was repeated three times, and Im‐cpl with 500 ng input was repeated four times. For low input RNA‐seq, Im‐cpl with 50, 20, 10 ng input and Sol‐RT with 100 ng input were repeated twice. Im‐cpl with 100 ng input was repeated four times. Reads and bases generated were obtained from Oxford Nanopore sequencing report. Mean and standard deviation were calculated using Excel. For Poly(A) tail length analysis, reads from two replicates were pooled for poly(A) tail length analysis. Sample sizes of the four protocols used for poly(A) tail length analysis are: Sol‐seq 253987 reads, Sol‐cpl 1334900 reads, Im‐cpl 2331366 reads, and Sol‐RT 747482 reads. The mean and median poly(A) tail lengths were calculated for each protocol and were used for comparison between different protocols. Plotting of poly(A) tail length distributions was done using R studio 1.3.1073. For Alignment length analysis, sample sizes of alignment length analysis are: Im‐cpl 500: 250249 primary alignments, Im‐cpl 100: 205448 primary alignments, Sol‐RT 500 116680 primary alignments, and Sol‐RT 100 41892 primary alignments. The mean and median alignment lengths were calculated for each dataset. Plotting of alignment length distributions was done using R studio 1.3.1073. For Gene expression analysis: The featureCount v2.0.1 was used to quantify reads that map to individual genes in the *L. monocytogenes* reference genome from the minimap2 alignments.^[^ ^28^ ^]^ The gene counts were normalized by gene lengths, and the total number of aligned reads. The normalized read counts TPM were used for pairwise comparison and correlation plots between different methods. Pearson correlation was used to measure the linear correlation between two datasets. 1097 common genes between Im‐cpl 500 (1/3) and Sol‐RT 500, 1327 common genes between Im‐cpl 500 (total) and Sol‐RT 500, and 274 common genes between Im‐cpl 100 and Sol‐RT 100 were used respectively for pairwise comparison and Pearson correlation coefficiency calculation. The raw gene count matrices of two technical replicates of the Im‐cpl and the Sol‐seq datasets were used for differential gene expression analysis using the Bioconductor package DESeq2 v3.12 in R Studio v1.3.1073.^[^ ^29^ ^]^ DESeq2 uses negative binomial distribution to make estimates and perform statistical inferences on differences.^[^ ^29^ ^]^ There were 1083 genes compared for differential expression, and none of the genes was found differentially expressed at a *p*‐value of 0.05. For per‐gene sequencing coverage analysis, sample sizes of gene sequencing coverage analysis are Im‐cpl 500: 1319; Im‐cpl 100: 1081; Sol‐RT 500: 1528, and Sol‐RT 100: 336 (Table 3). The mean and median sequencing coverage were calculated for each dataset. Plotting of sequencing coverage distributions was performed using R studio 1.3.1073.

## Conflict of Interest

The authors declare the following conflict of interest(s): Y.F., L.S., A. Z., Z. S., and M. X. are employees of New England Biolabs, Inc., a commercial supplier of reagents for molecular biology and inventors of a US patent related to this work (No. 17/018,862, filed 11 September 2020).

## Supporting information

Supporting Information

## Data Availability

The data that support the findings of this study are openly available in European Nucleotide Archive at https://www.ebi.ac.uk/ena/browser/home, reference number PRJNA683132.
