# Supplementary material for: Nanopore Whole Transcriptome Analysis and Pathogen Surveillance by a Novel Solid‐Phase Catalysis Approach
Source: Adv Sci (Weinh). 2021 Nov 27;9(3):2103373. doi: 10.1002/advs.202103373 (PMC8787394; doi:10.1002/advs.202103373)
Supplement: Supplementary file 1 — Supporting Information [file ADVS-9-2103373-s001.pdf]

WILEY-VCH

ADVANCED SCIENCE
Open Access

Supporting Information

for *Adv. Sci.*, DOI: 10.1002/advs.202103373

Nanopore Whole Transcriptome Analysis and Pathogen Surveillance by A Novel Solid-Phase Catalysis Approach

Yi Fang, *Amogh Changavi*, *Manyun Yang*, *Luo Sun*, *Aihua Zhang*, *Daniel Sun*, *Zhiyi Sun**, *Boce Zhang**, and *Ming-Qun Xu**

Supporting Information

Nanopore Whole Transcriptome Analysis and Pathogen Surveillance by A Novel Solid-Phase Catalysis Approach

Yi Fang, Amogh Changavi, Manyun Yang, Luo Sun, Aihua Zhang, Daniel Sun, Zhiyi Sun, Boce Zhang*, Ming-Qun Xu**

Table S1. Poly (A) length analysis from different protocols with 500 ng input.

Protocol	Mean	Median
Sol-seq	75.1	60.1
Sol-cpl	29.4	22.6
Sol-RT	100.5	94.9
Im-cpl*	24.8	19.7

* Im-cpl data analysis used combined reads from three flow cell sequencing run datasets of a single 500 ng *L. monocytogenes* RNA library.

Table S2. RNA-seq read qualities using low-input Im-cpl protocol without rRNA removal.

	RNA low input amount (ng)			
	Im-cpl 100	Im-cpl 50	Im-cpl 20	Im-cpl 10
Mapped Rate (%)	99.0	99.1	98.7	98.4
Mean read quality	10.1	10.0	10.2	10.3
Median read quality	10.1	10.1	10.3	10.4

* Analysis was performed using RNA-seq data from *L. monocytogenes* RNA libraries with the low-input amounts (10, 20, 50 or 100 ng).

Table S3: Internal data comparison using various library preparation protocols.

	Unique genes	Common genes	Total genes	Total genes (Average)	Common genes percentage
Im-cpl 500	319<br>298	1000	1319<br>1298	1309	76 %
Sol-RT 500	281<br>330	1198	1479<br>1528	1504	80 %
Im-cpl 100	432<br>177	649	1081<br>826	954	69 %
Sol-RT 100	109<br>320	227	336<br>547	442	55 %

* Assessment was performed using RNA-seq data from replicate libraries prepared by each protocol.

Table S4. Comparison of Im-cpl and Sol-RT protocols using synthetic spike-in transcripts

	# Transcript expected	Im-cpl mapped reads (# Transcript identified)	Sol-RT mapped reads (# Transcript identified)
ERCC	38#	5390 (37)	26 (12)
Long SIRVs	15	528 (15)	6 (5)

* Lexogen's SIRV-Set 4 standard was used for validation of Im-cpl or Sol-RT protocols for preparation of 50 ng RNA input community samples. The Im-cpl displays much higher recovery of the synthetic spike-in transcripts, including External RNA Controls Consortium (ERCCs) and long SIRVs.

# The number of expected transcripts was calculated based on the relative abundance of synthetic spike-in transcripts in the starting material and the total number of sequencing reads. Only spike-in transcripts whose expected read count is greater than 1 are counted.

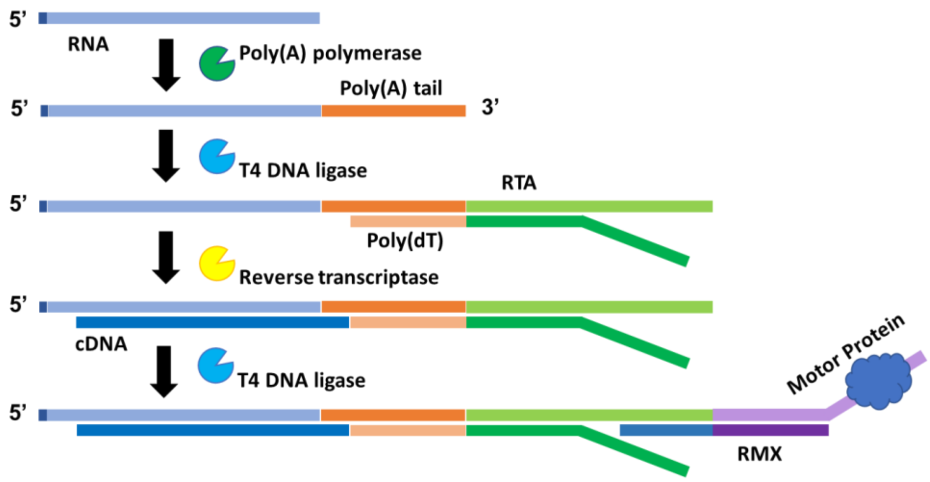

Figure S1. Illustration of RNA library preparation from non-polyadenylated RNA for Nanopore direct RNA-seq.

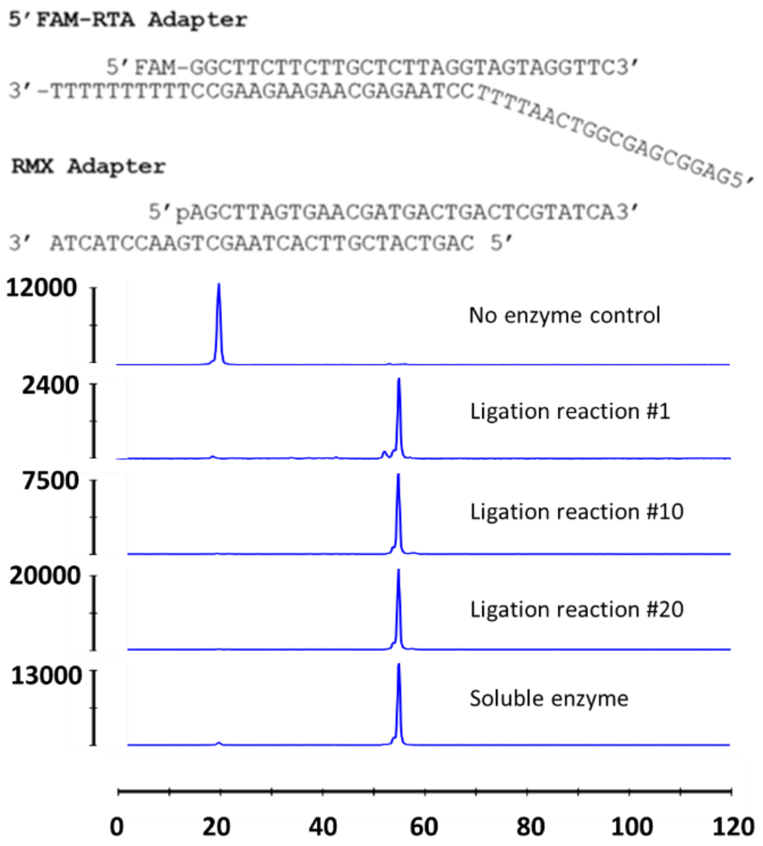

Figure S2. Adaptor ligation activity and reusability of immobilized T4 DNA Ligase. 300 units of T4 DNA ligase immobilized onto magnetic beads (60 units/µL) was utilized to perform 20 repeated ligation reactions. In each reaction cycle, two synthetic adaptors, 5’FAM-RTA (100 nM) and RMX (150 nM) were added to the immobilized ligase and incubated in 25 µL 1x Quick Ligase Buffer (QL) at 25 °C for 10 min; the enzyme-bearing beads were pelleted on magnetic rack; the product-containing supernatant was removed from the vial and transferred for capillary electrophoresis analysis; and the pelleted beads were quickly washed 5 times with 50 µL 1x QL in conjunction with micro-centrifugation in preparation for the next adaptor ligation cycle. The data (reactions #1, #10, and #20 shown) demonstrate efficient ligation in 20 consecutive ligation reactions, which is indicative of the reliability and reproducibility of immobilized T4 DNA ligase. The performance over these twenty reactions matches the results observed in a single reaction with soluble T4 DNA ligase (2000 units, NEB, M0202) for the same substrates.

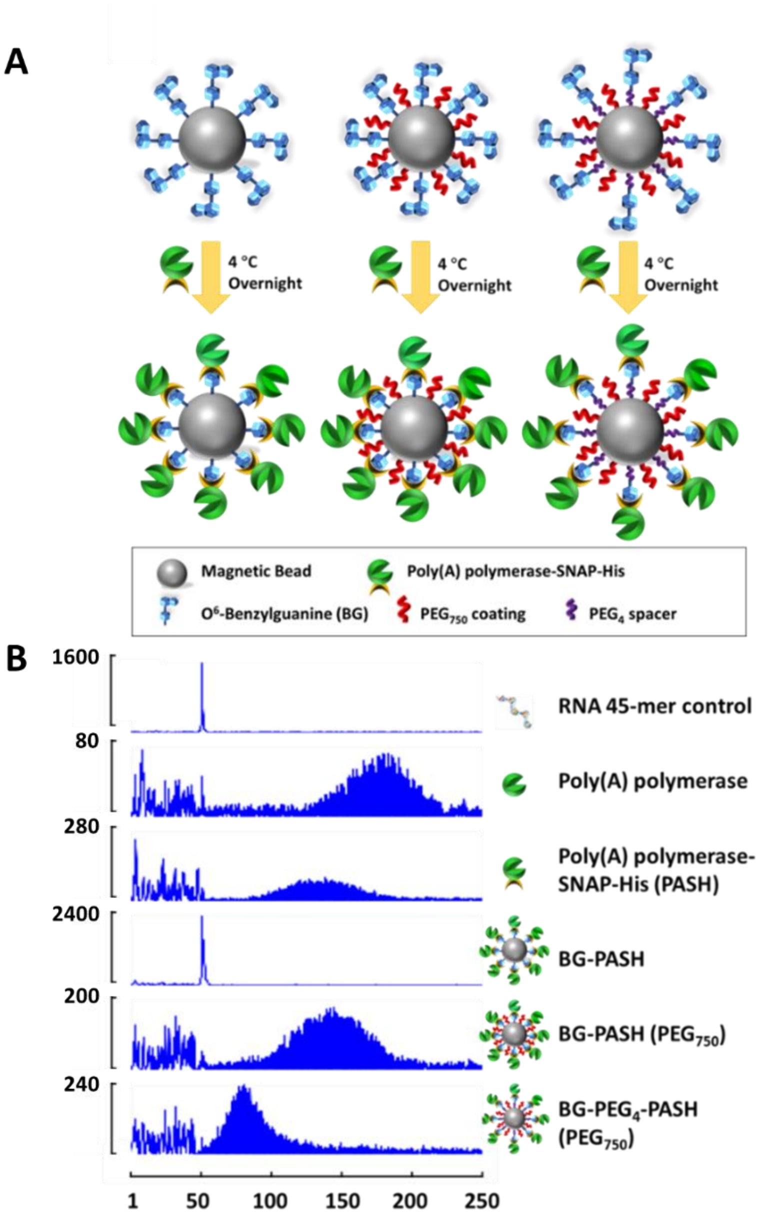

Figure S3. Enzyme immobilization strategies and poly(A) tailing activity. (**A**) Schematic illustration of different immobilization strategies for SNAP-tagged *E. coli* poly(A) polymerase (PASH) using PEG_{750} coating of magnetic microbead surface, and PEG_4 as a spacer between bead surface and a SNAP-tagged enzyme molecule. (**B**) Capillary electrophoresis analysis of the enzymatic activity of two soluble forms, untagged PAP and PASH fusion protein, and the immobilized PASH forms using different strategies.

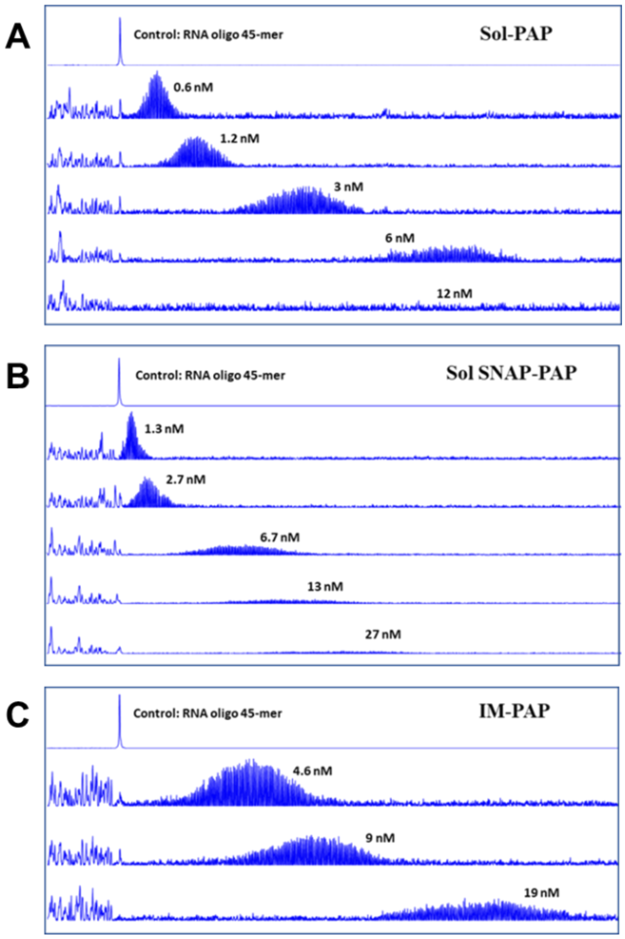

Figure S4. Characterization of poly(A) tailing activity of various forms of *E. coli* poly(A) polymerase. RNA oligo 45-mer, 5'FAM-AAGGAGAAGAGAAGAGGAAGAAAACUAACA CAGGAGAGAGAAGG A was treated with different concentrations of soluble untagged PAP (Sol-PAP, NEB M0276), soluble SNAP-tagged PAP, or immobilized SNAP-tagged PAP (Im-PAP). 10 ul-reactions were set up by mixing the following components: 6 µL nuclease-free water, 1 µL 10X poly(A) polymerase reaction buffer (NEB), 1 µL 10 mM ATP, 0.5 µL RNase inhibitor, 1 µL 1 µM RNA 45-mer oligo and 0.5 µL poly(A) polymerase (diluted in different concentrations). The mixtures were incubated at 37 °C for 20 min. Each reaction was quenched by adding 10 µL of 50 mM EDTA with 0.7 % Tween-20. The reactions were further diluted 10 times to a final volume of 200 µL (to a final 5 nM of RNA-45mer concentration) for capillary electrophoresis (CE) analysis. The CE data show that the 3'poly(A) extension length is dependent on enzyme concentration, and IM-PAP is capable of proficient 3' poly(A) tailing.

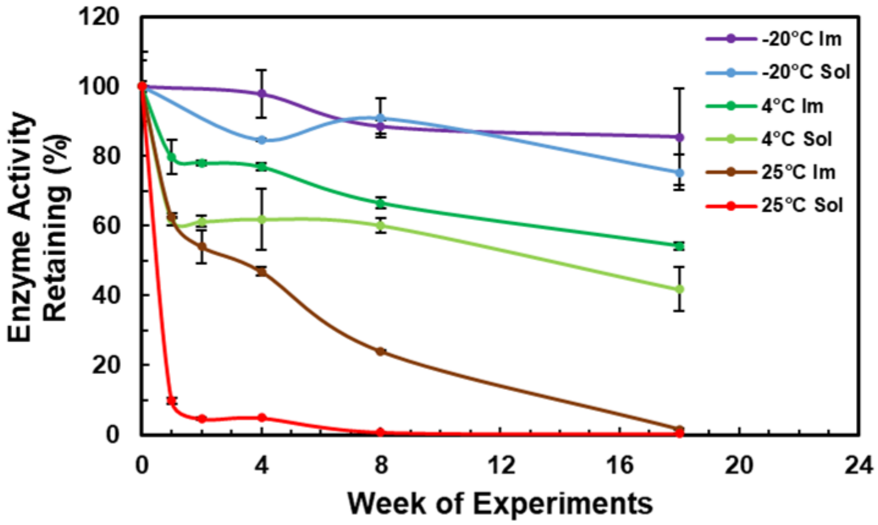

Figure S5. Comparison of stability of soluble and immobilized *E. coli* poly(A) polymerase (PAP). Poly(A) tailing activity of soluble and immobilized PAP (Sol and Im, respectively) was assayed using 5’FAM-RTA RNA 35-mer substrate following storage under various temperatures indicated. The immobilized PAP displayed the same or better stability compared to its soluble counterpart.

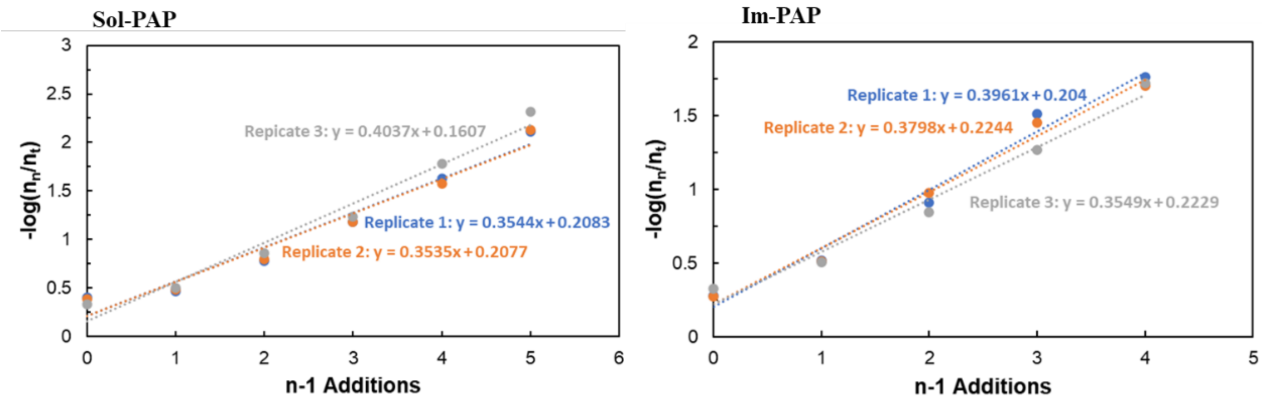

$$\log(n_I/n_T) = (n-1)\log P_I + \log(1-P_I).$$

	P_I	Processivity (Pr)
Soluble PAP	0.392 ± 0.034	1.647 ± 0.090
Immobilized PAP	0.407 ± 0.017	1.686 ± 0.048

Figure S6. Assessment of processivity of soluble and immobilized *E. coli* poly(A) polymerase (PAP). Processivity assay was carried out for soluble and immobilized PAP (Sol-PAP and Im-PAP, respectively) using 5'FAM-RTA RNA 35-mer substrate according to the method described by Hippel et al. (HIPPEL, P.H., Fairfield, F.R. and Dolejsi, M.K., 1994. On the processivity of polymerases. Annals of the New York Academy of Sciences, 726(1), pp.118-131.). – log(nI/nt) was plotted against n-1 additions of nucleotides based on capillary electrophoresis analysis of 3' poly(A) addition. Processivity (Pr), determined as 1/(1-PI), measures the ability of the enzyme to catalyze consecutive reactions without releasing its substrate. Soluble and immobilized PAP exhibit essentially the same processivity, indicating SNAP-tagging and immobilization onto these magnetic microbeads do not affect the kinetic property of PAP under the assay conditions.

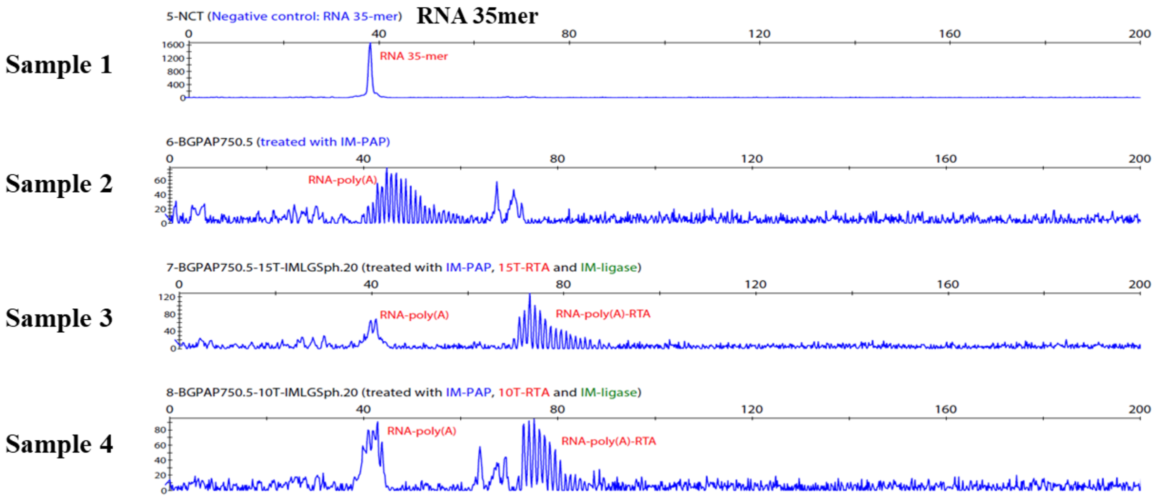

Figure S7. Poly(A) tailing and adaptor ligation activity from immobilized poly(A) polymerase (Im-PAP) and immobilized T4 DNA Ligase (Im-Ligase). Synthetic 5'FAM-RTA RNA 35-mer (RNA-35mer, 5'FAM-AAGAAGGAAGAAAACUAACACAGGAGAGAGAAGGA) was used as the substrate for poly(A) tailing with IM-PAP and subsequent ligation with RTA adaptor catalyzed by IM-Ligase. Four samples were examined by capillary electrophoresis analysis (from top to bottom): Sample 1, untreated FAM-labeled RNA substrate showing a distinct peak; sample 2, FAM-labeled RNA substrate treated by immobilized Poly(A) polymerase; Sample 3, Sample 2 treated with immobilized T4 DNA ligase and RTA-poly(dT)15, RTA adaptor with 3'15T. Sample 4, Sample 2 treated with immobilized T4 DNA ligase and RTA-poly(dT)10, RTA adaptor with 3'10T. A bell-shaped peak in Sample 2, RNA-poly(A) represents an addition of 3' poly(A) tails of various lengths to the RNA substrate (Sample 1). Ligation of an RTA adaptor to the poly(A) tailed products generated higher molecular mass products, RNA-poly(A)-RTA, resulting in a shift of the bell-shaped peak to the right.

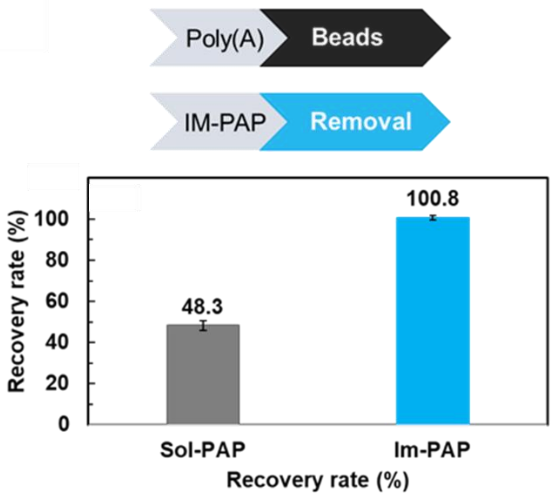

Figure S8. Comparison of the recovery rate of RNA library preparations. 500 ng of *L. monocytogenes* RNA was treated by soluble poly(A) polymerase followed by a step of SPRI bead-based purification or immobilized-poly(A) polymerase without bead-based purification.

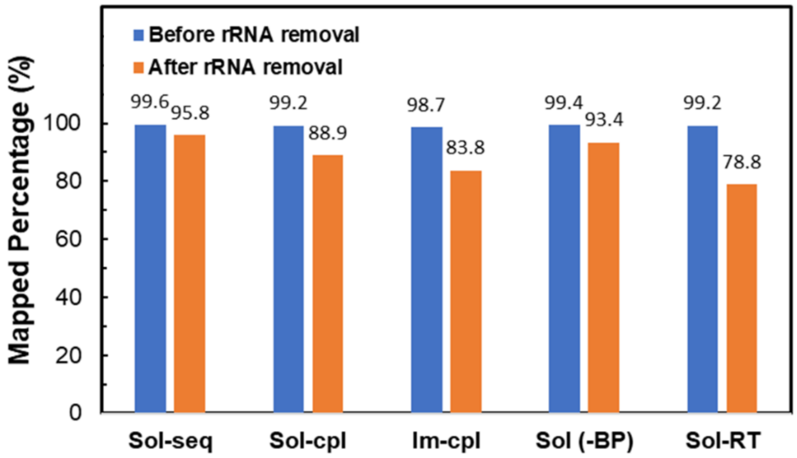

Figure S9. Percentages of mapped reads to the *L. monocytogenes* genome of different RNA library preparation methods before and after rRNA sequence removal. 500 ng of *L. monocytogenes* RNA was used for all experiments.

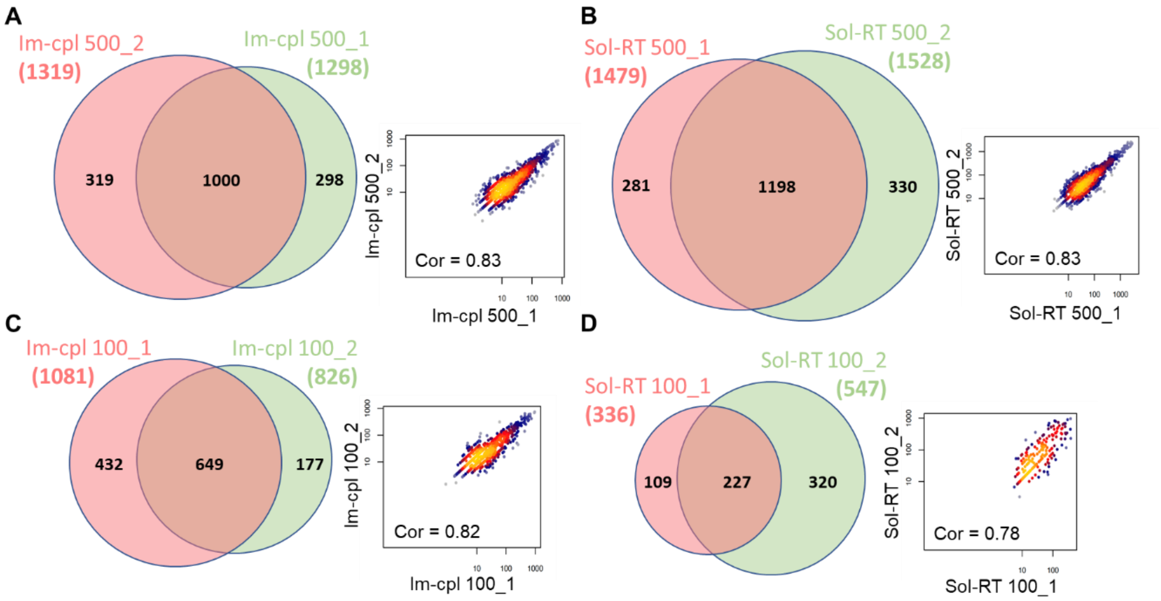

Figure S10. Venn Diagrams and Transcripts Per Million (TPM) correlations of two replicates of Im-cpl and Sol-RT library preparation protocols and different *L. monocytogenes* RNA input amounts. (**A**) Im-cpl protocol with 500 ng RNA input. (**B**) Sol-RT protocol with 500 ng RNA input. (**C**) Im-cpl protocol with 100 ng RNA input. (**D**) Sol-RT protocol with 100 ng RNA input.

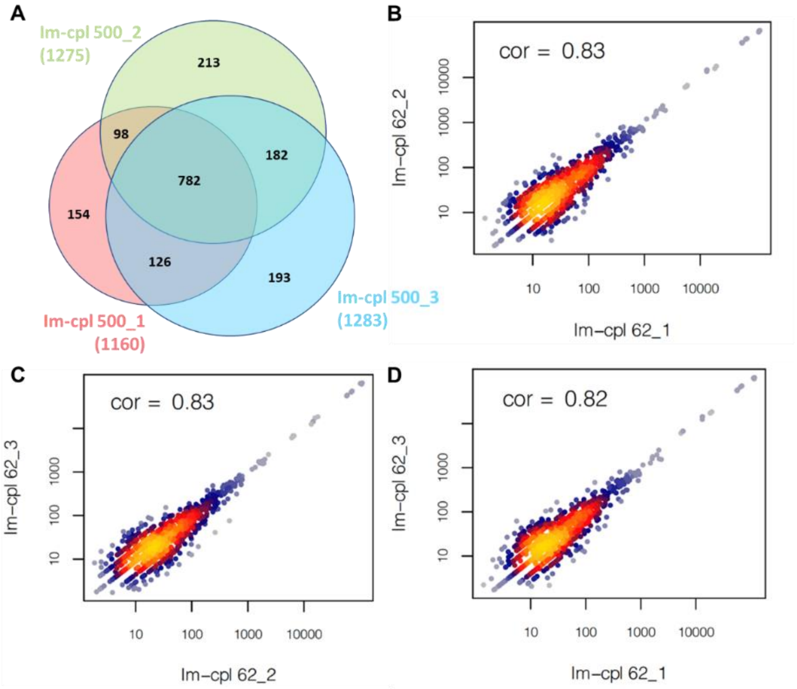

Figure S11. Analysis of a single Im-cpl library of 500 ng RNA input using three flow cells. **(A)** Venn Diagrams of three flow cell sequencing run from a single library preparation using Im-cpl protocol for 500 ng *L. monocytogenes* RNA input. (**B,C,D**) Pair-wise comparisons of the Transcripts Per Million (TPM) and Pearson correlation coefficients of common genes between the three singular sequencing runs of the Im-cpl 500 library.

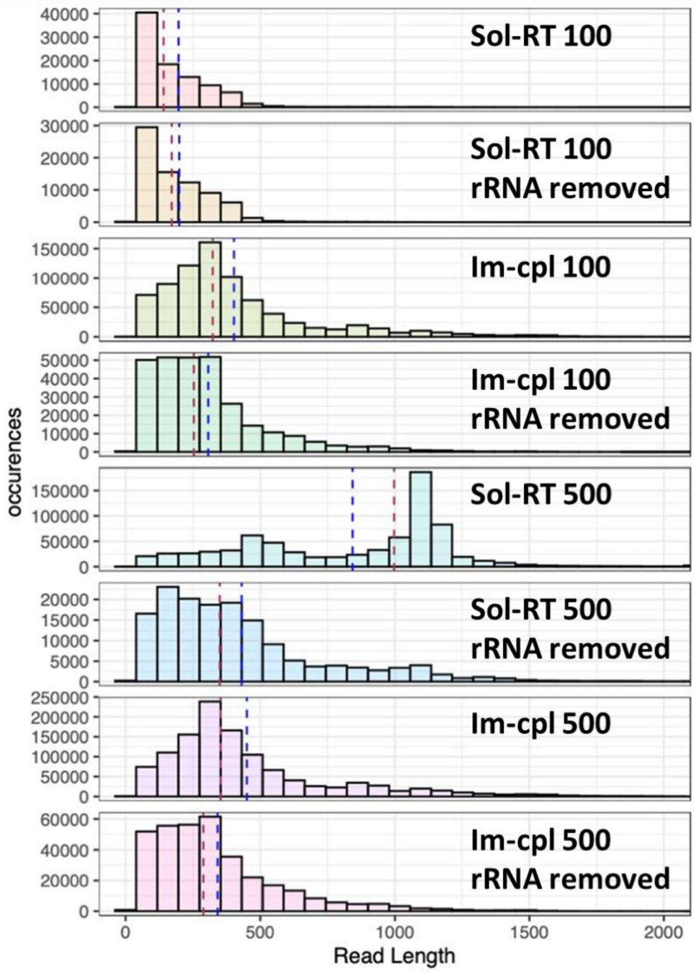

Figure S12. Read length distribution of the libraries of the Im-cpl and Sol-RT protocols with both 500 ng and 100 ng of low input RNA sequencing. Blue and red dash lines denote mean and medium read lengths.

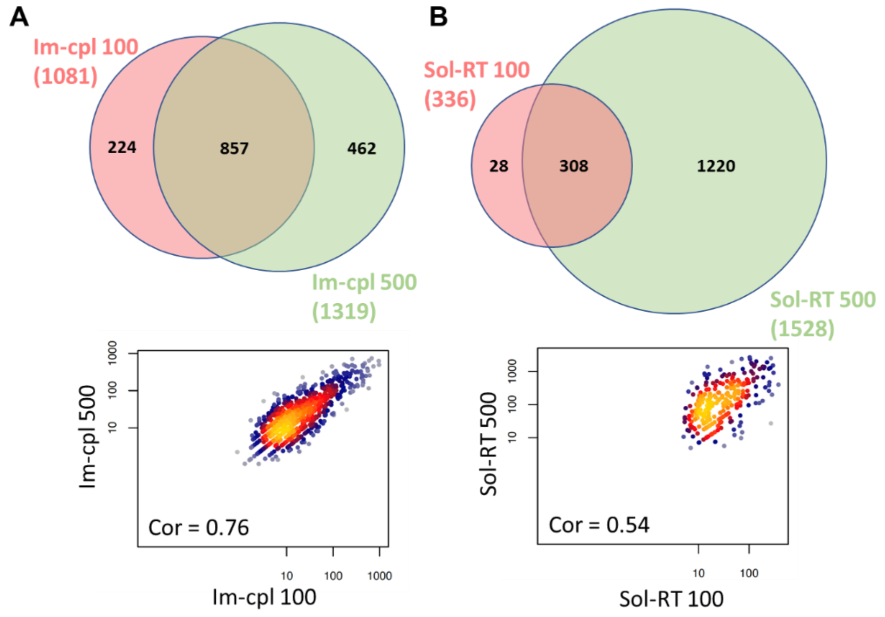

Figure S13. Comparison of low and high input Im-cpl and Sol-RT protocols. Shown are Venn Diagrams and transcripts per million (TPM) correlations of RNA-seq of Im-cpl and Sol-RT protocols using *L. monocytogenes* total RNA with low and high input amounts. (**A**) The Venn Diagram comparison (upper) and TPM (lower) of Im-cpl protocol using 100 ng and 500 ng of RNA input. (**B**) The Sol-RT's Venn Diagram comparison (upper) and TPM (lower) using 100 ng and 500 ng of RNA input.

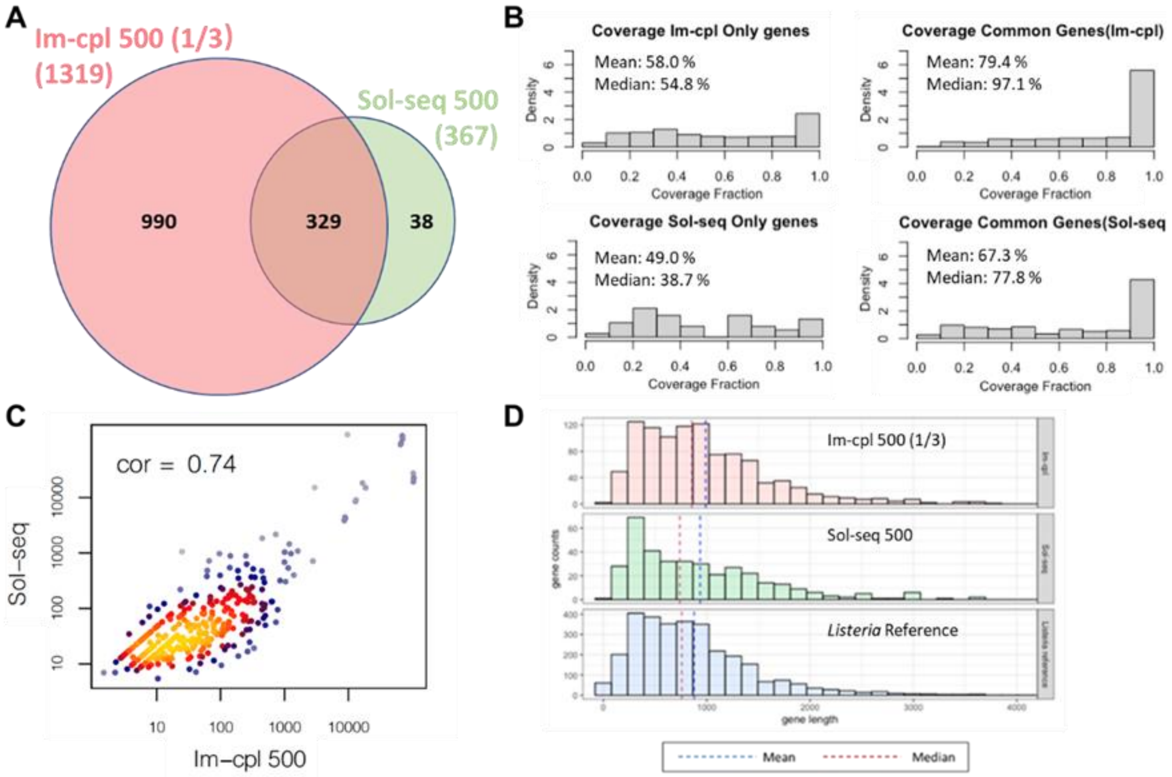

Figure S14. Comparison of genes identified from Im-cpl and Sol-seq protocols. (**A**) Venn Diagram of genes identified from Im-cpl (one-third of RNA library was loaded) and Sol-seq protocol. Both protocols omit reserve transcriptase treatment. (**B**) The coverage of genes identified from Im-cpl and Sol-seq protocols for both common and unique genes that are covered by each protocol. (**C**) Pearson correlation of Im-cpl and Sol-seq protocol. (**D**) Distribution of gene counts at different gene lengths from Im-cpl (one-third of RNA library was loaded) and Sol-seq protocol in comparison to the distribution of the *L. monocytogenes* reference genome.

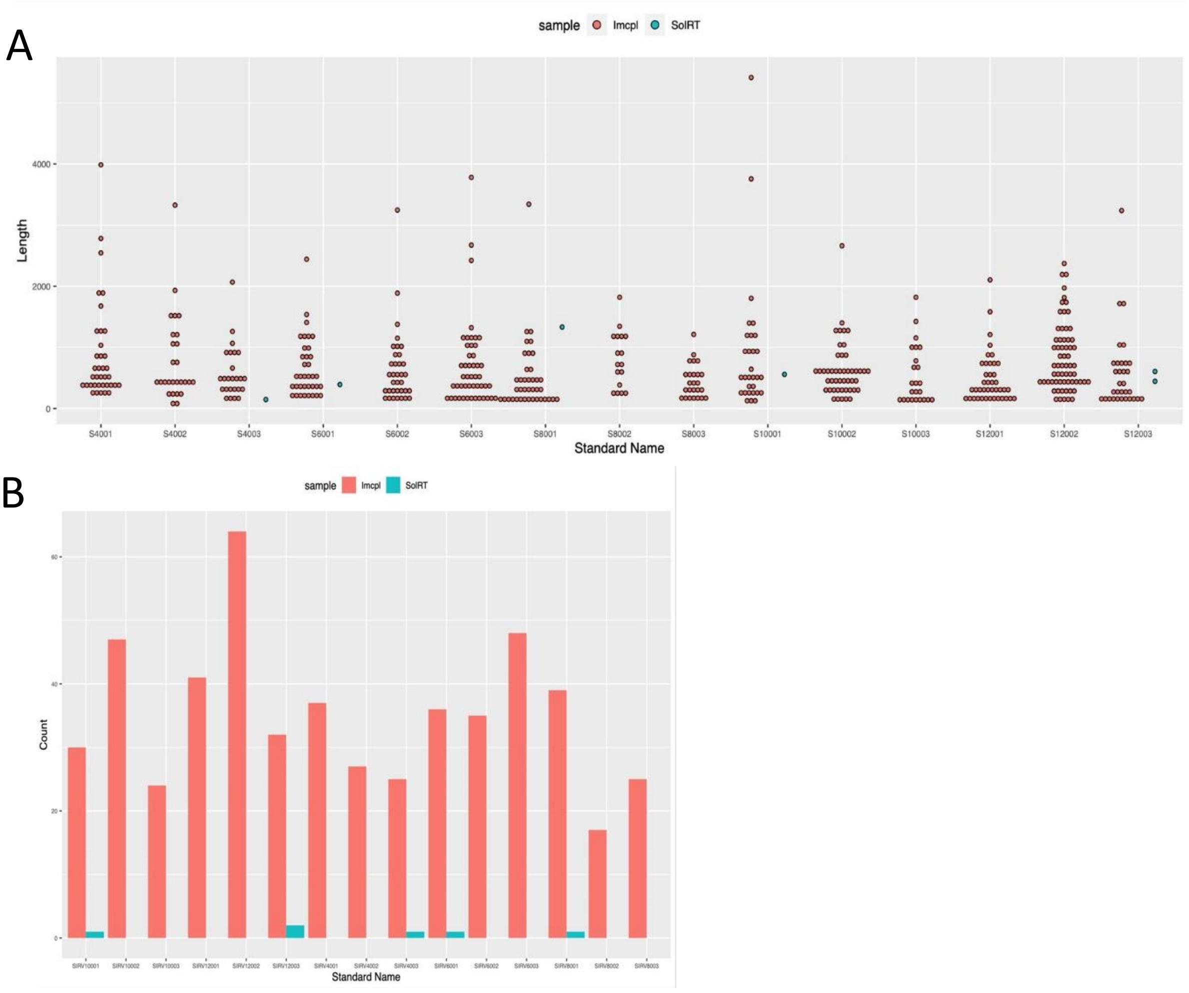

Figure S15. Comparison of Im-cpl and Sol-RT protocols by validation of long transcript standards. 0.5 ng of Lexogen's synthetic transcript mixture, SIRV Set 4, added to 50 ng community RNA mixture (containing 39 ng *E. coli* O157:H7, 10 ng *Salmonella* Enteritidis, and 1 ng *L. monocytogenes* transcriptome (Table 2), was processed by either Im-cpl or Sl-RT protocol and sequenced with a R9.4 flow cell. (**A**) Length distribution of the reads mapped to long SIRVs. These long synthetic transcripts consist of 5 different size subgroups, with each containing three species. (**B**) Mapped read counts of SIRV Set 4 long transcript standards. For low-input RNA amount, the Im-cpl protocol results in a significant more uniform recovery of all 15 transcripts.
